# Advances in Nanostructured Electrodes for Solid Oxide Cells by Infiltration or Exsolution

**DOI:** 10.3390/ma18081802

**Published:** 2025-04-15

**Authors:** Mingyue Dai, Futao Li, Shujuan Fang, Dedong He, Jichang Lu, Yu Zhang, Xiaohua Cao, Jiangping Liu, Dingkai Chen, Yongming Luo

**Affiliations:** 1 Faculty of Chemical Engineering, Kunming University of Science and Technology, Kunming 650500, China; dmy202503@163.com (M.D.); lft12315@163.com (F.L.); yoloim@163.com (S.F.); kust202503@163.com (D.H.); xiaohuacao@kust.edu.cn (X.C.); 2 The Innovation Team for Volatile Organic Compounds Pollutants Control and Resource Utilization of Yunnan Province, Kunming 650500, China; lujichangc7@kust.edu.cn (J.L.); liujiangping@kust.edu.cn (J.L.); 3 Key Laboratory of Yunnan Province for Synthesizing Sulfur-containing Fine Chemicals, and The Higher Educational Key Laboratory for Odorous Volatile Organic Compounds Pollutants Control of Yunnan Province, Kunming 650500, China; 4 Faculty of Environmental Science and Engineering, Kunming University of Science and Technology, Kunming 650500, China; zhangyu_kust@163.com

**Keywords:** energy conversion, solid oxide cells (SOCs), nanocomposite electrodes, infiltration, metal nanoparticle exsolution

## Abstract

Solid oxide cells (SOCs) are highly efficient and versatile devices capable of utilizing a variety of fuels, presenting promising solutions for energy conversion and renewable resource utilization. There is an urgent need for the strategic design of robust and high-efficiency materials to enhance both conversion and energy efficiencies before SOCs can be applied for large-scale industrial production. Nanocomposite electrodes, especially those fabricated through infiltration and metal nanoparticle exsolution, have emerged as highly active electrocatalytic materials that significantly improve the performance and durability of SOCs. This review systematically summarizes and analyzes recent advances in the nanoscale architecture of electrode materials fabricated via common nanoengineering strategies, including infiltration and in situ exsolution, with applications in CO_2_/H_2_O reduction, hydrocarbon electrochemical oxidation, solid oxide fuel cells, and reversible operation. Finally, this review highlights existing bottlenecks and promising breakthroughs in common nanotechnologies, aiming to provide useful references for the rational design of nanomaterials for SOCs.

## 1. Introduction

**Figure 1:**
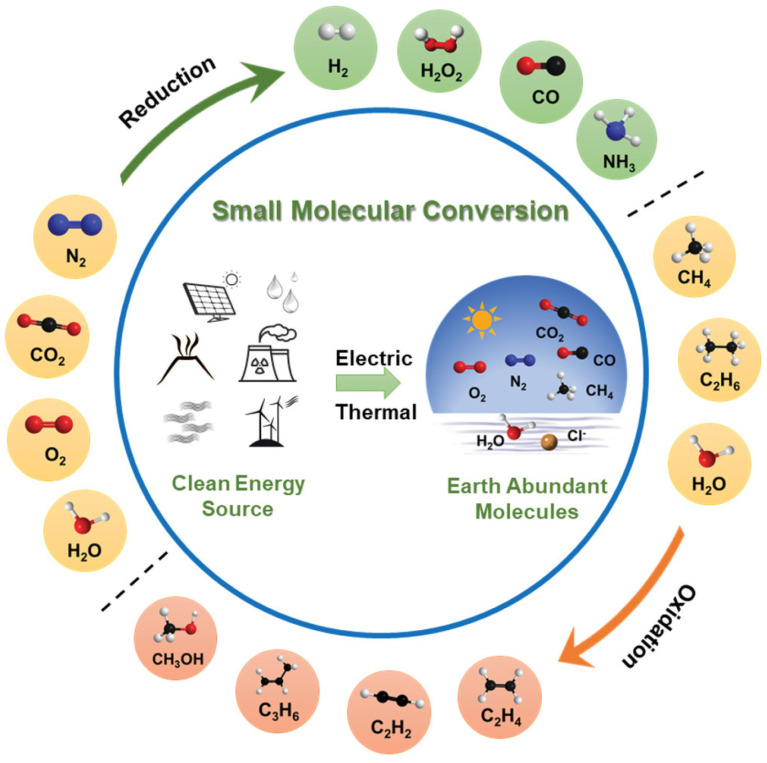
Conversion of small molecules by thermal or electrochemical catalytic processes utilizing renewable energy sources. Ref. [6]. Reproduced with permission from [6].

Energy is a fundamental resource essential for human survival and economic development. Currently, fossil fuels continue to dominate the global energy landscape. The accelerating depletion of fossil fuel reserves has led to a dual crisis: energy scarcity and environmental degradation [1]. Fossil fuel production and consumption generate significant carbon dioxide emissions, thereby exacerbating global warming and its adverse effects. In response, global efforts have intensified to mitigate climate change, with long-term goals focused on achieving carbon peaking and carbon neutrality [2,3,4]. Consequently, it is imperative to develop a diversified energy system that integrates renewable energy sources and advanced clean technologies for efficient petrochemical resource utilization, as illustrated in Figure 1. To date, considerable progress has been made in advancing sustainable energy harvesting technologies, including solid oxide cells (SOCs), direct carbon fuel cells, wind energy systems, and solar panels [5].

**Figure 2:**
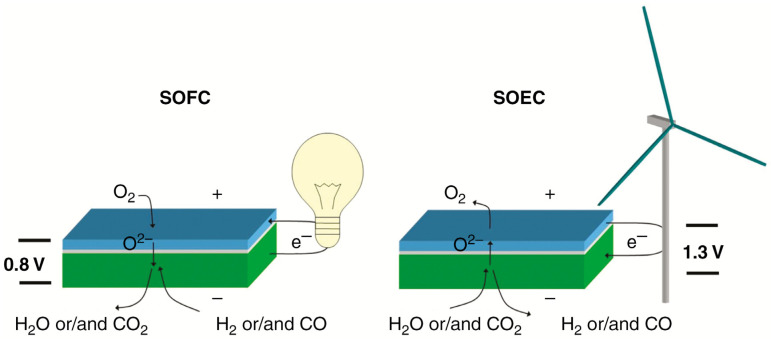
Schematic illustration of the principle of solid oxide cells (SOCs) in SOFC and SOEC modes of operation. The blue top layer represents the porous oxygen electrode, the white intermediate layer is the dense oxide-ion-conducting electrolyte, and the green bottom layer depicts the porous fuel electrode along with the porous structural support of the cell. The typical operating temperature ranges from 750 to 800 °C [20]. Reproduced with permission from [20].

Solid oxide cells (SOCs) represent a highly efficient energy conversion technology capable of stable operation in dual modes. In the solid oxide fuel cell (SOFC) mode, when hydrogen (H_2_) is used as the fuel, oxygen from the air enters the cell through the cathode. At the cathode, O_2_ molecules diffuse and are reduced to oxygen ions (O_2_ + 4e^-^ → 2O^2-^), which subsequently traverse the electrolyte to reach the anode. At the anode, the hydrogen fuel reacts with the migrating oxygen ions (2H_2_ + 2O^2-^ → 2H_2_O + 4e^-^). If there is an external circuit connecting the electrodes, electrons flow from the anode to the cathode, maintaining a steady supply of oxygen ions (O^2-^) in the electrolyte while ensuring overall charge neutrality. This process ultimately generates electrical energy through the electrochemical oxidation of the fuel. Conversely, in the solid oxide electrolysis cell (SOEC) mode, renewable electricity is utilized to decompose water molecules (H_2_O) and carbon oxides into H_2_ or other fuels and O_2_. In the case of H_2_O, water molecules diffuse to the reaction site at the cathode–electrolyte interface, where they dissociate into hydrogen gas and oxygen ions (H_2_O + 2e^-^ → H_2_ + O^2-^). The hydrogen gas diffuses to the surface of the cathode for collection, while the oxygen ions migrate through the dense electrolyte to the anode. At the anode, the oxygen ions are oxidized back to molecular oxygen (2O^2-^ → O_2_ + 4e^-^) and released from the surface. This mechanism enables the efficient synthesis of fuels and chemicals using renewable energy sources [7]. as illustrated in Figure 2. Beyond clean energy conversion, SOCs offer significant potential for greenhouse gas utilization, contributing to carbon emission reduction. The all-solid-state structure of SOECs provides several advantages, including simplified electrode reactions, enhanced operational stability, high flexibility, extended service life, low material consumption, rapid kinetics, and superior energy efficiency, making SOECs economically valuable and efficient energy conversion systems [8,9]. Operating within a temperature range of 600–850 °C, SOECs align with the optimal conversion temperatures for various chemicals and fuels, significantly enhancing electrode reaction rates and catalytic activity. These attributes highlight the broad application prospects of SOCs in the renewable energy sector, attracting considerable research interest [10].

Despite the numerous advantages of solid oxide cells (SOCs), several critical challenges remain in their development. These challenges include system degradation during high-temperature operation and performance losses at lower temperatures [11]. Traditional methods for enhancing catalytic activity, such as atomic layer deposition [12,13], sol–gel techniques [14,15], and solution combustion methods [16], have demonstrated efficacy in improving material properties but often encounter stability issues. At elevated temperatures, material agglomeration can occur, leading to the formation of larger particles and subsequent performance degradation. Recent advancements have highlighted the significant potential of integrating nanostructures and related phenomena to enhance the performance of solid oxide cells (SOCs). The incorporation of nanomaterials into the electrode structure of SOCs markedly increases the available surface area for electrochemical reactions, thereby improving cell performance [17,18]. For example, coating the electrode surface with nanoscale particles or thin films creates a highly porous structure with an enlarged triple-phase boundary (TPB) surface area compared to conventional microstructured electrodes. This expanded surface area enhances the contact between the electrode material and the electrolyte, thereby accelerating ion and electron transport across the interface [19]. Additionally, nanostructured electrolytes have been developed to reduce ionic resistance and improve the ionic conductivity of the electrolyte material, facilitating faster oxygen ion transport through the electrolyte. These innovations not only minimize polarization losses but also significantly increase the overall efficiency of SOCs. Furthermore, researchers are actively exploring the design of novel nanocomposite electrodes to ensure both durability and superior performance.

Among various strategies for promoting long-term operation, infiltration remains one of the most effective methods for fabricating continuous nanolayers on porous electrodes. This method involves infiltrating a precursor solution containing active materials into the porous electrode scaffold, followed by calcination to form composite electrodes [21]. Solution infiltration minimizes mismatches in thermal expansion coefficients and mitigates adverse chemical interactions between materials. Additionally, it enhances the triple-phase boundary length and increases the specific surface area of electrodes. Functional material infiltration significantly improves the catalytic capacity, conductivity, and long-term stability of electrodes [22]. Furthermore, metal nanoparticle exsolution on perovskite surfaces offers a robust approach to mitigating particle agglomeration at high temperatures. In situ exsolution of metal nanoparticles generates highly active nanoparticles and oxygen-vacancy-enriched perovskite structures. Moreover, exsolution exhibits a certain degree of reversibility during redox cycling, contributing to improved stability. It is also important to note that the regeneration efficiency of nanoparticles is strongly correlated with the redox properties of perovskite oxides [23]. Consequently, in situ exsolved nanoparticles demonstrate enhanced catalytic activity and stability during electrolysis. At present, advanced manufacturing techniques, such as atomic layer deposition (ALD), pulsed laser deposition (PLD), and sputtering, are also being explored for developing advanced SOC electrodes [24,25,26]. Pulsed laser deposition (PLD) is a low-temperature fabrication technique particularly well-suited for the development of micro-solid oxide fuel cells (micro-SOFCs) based on multilayer thin films. The fundamental principle of this technology involves irradiating a target material with high-power laser pulses, inducing its evaporation and ionization through an “ablation” process. This generates a high-brightness plasma plume, enabling the rapid transfer of material from the target to the substrate, thereby facilitating the formation of a thin film [27]. Key advantages of PLD include its potential for automation and high technical reliability. Sputtering technology operates on the principle that high-energy plasma or gas particles bombard the surface of a solid material, causing surface particles to be ejected. In this process, the substrate is positioned opposite to the target. To establish a self-sustaining plasma, argon gas is typically introduced into a vacuum chamber, where an electric field accelerates argon ions to bombard the target material. Atoms of the target are sputtered by the plasma/gas particles and subsequently move toward the substrate, depositing a thin film [28]. Despite their effectiveness, these processes have certain limitations. For instance, the requirement for high substrate temperatures or subsequent high-temperature annealing to enhance film crystallinity may result in interfacial reactions or thermal stress issues. Additionally, achieving uniform dispersion of nanoparticles during direct deposition of dense thin films remains challenging, limiting active sites. Furthermore, reliance on expensive equipment and targets, coupled with high maintenance costs, hinders industrial scalability. In contrast, infiltration and exsolution strategies offer distinct advantages over the aforementioned advanced processes. These strategies enable precise control over the loading and distribution of nanocatalysts, ensuring high activity and a large specific surface area of catalytic sites. Moreover, they are cost-effective and scalable, making them highly promising for industrial applications. Therefore, from the perspective of commercial feasibility and industrial applications, the potential availability of nanostructured electrode manufacturing technology primarily depends on manufacturing cost and scalability.

Overall, the maturity of SOCs for efficient electricity generation from chemical energy or chemical synthesis from electricity necessitates the development of low-cost, highly stable, and enhanced electrocatalytic electrode materials to reduce construction and operational costs. Numerous excellent reviews have been published on the development of SOFCs and SOECs, focusing on materials [29], microstructures [30,31], interfaces [32,33,34], and systems [20,35]. In the context of extensive research over the past decade, this review aims to provide a comprehensive overview of nanostructured electrode materials for SOCs. Particular emphasis is placed on the infiltration and exsolution techniques for developing advanced electrodes. Additionally, the advancements in symmetrical and reversible SOCs for energy storage applications are summarized. Finally, this review evaluates constructive perspectives to achieve promising breakthroughs in advanced solid oxide cells (SOCs) through common nanotechnologies. The aim is to reveal new opportunities in both fundamental research and the broad commercialization of SOCs.

## 2. Infiltration

**Figure 3:**
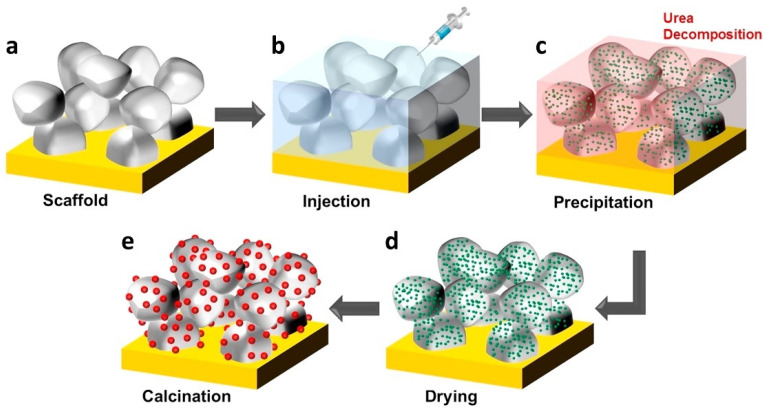
Schematic illustration of the synthesis of nanocatalysts in a pre-sintered porous electrode via a wet chemical infiltration process: (**a**) preparation of the scaffold, (**b**) injection of the chemical solution, (**c**) precursor precipitation induced by urea decomposition, (**d**) solvent removal, and (**e**) calcination. Ref. [40]. Reproduced with permission from [40].

Infiltration serves as a pivotal technology for optimizing material performance or extending functionality by deeply integrating precursors with porous materials. In the context of solid oxide cells (SOCs), the typical infiltration process is illustrated in Figure 3. Here, external functional phases (nanocatalysts) are introduced into a preconstructed SOC electrode skeleton, which is subsequently sintered at low temperatures to preserve its nanoscale characteristics. Prior to infiltration, the electrode skeleton is pre-sintered on the electrolyte surface and engineered to possess an adequate pore structure. Subsequently, a solution containing the functional phase precursor, with precisely controlled loading, is injected into the porous skeleton via a syringe. The permeation process is often repeated multiple times to achieve the desired loading. Following settling, drying, and calcination, the nanocatalysts are formed and securely anchored to the electrode skeleton [36]. The tortuosity factor (τ) of the infiltrated phase has emerged as a critical parameter for enhancing electrode performance through the optimization of transport pathways and three-phase interfaces. A higher τ value results in increased mass transport resistance and diminished diffusion efficiency. τ exhibits strong correlations with the particle size, morphology, distribution uniformity of the infiltrated phase, and the particle size of the backbone structure [37]. Reducing τ can effectively decrease polarization resistance while simultaneously extending the triple-phase boundary (TPB) length to enhance reaction kinetics [38].

The advancement of infiltration techniques has facilitated the deposition of both discontinuous (discrete nanoparticle layers) and continuous (dense coatings) catalysts on state-of-the-art electrodes such as LSM (lanthanum strontium manganite) and LSCF (lanthanum strontium cobalt ferrite). These modifications have demonstrably enhanced the surface electrocatalytic activity and operational stability of electrodes [39]. Infiltration involves introducing nanoparticles—comprising metals (Section 2.1), perovskites (Section 2.2), and other functional materials (Section 2.3)—onto electrode surfaces to form composite electrodes. This method significantly improves catalytic performance by increasing the density of reaction sites, ensuring better material utilization, and enhancing long-term stability. Embedding active nanoparticles into the porous electrode structure not only boosts the number of active sites but also promotes more efficient material usage and durability. Table 1, Table 2 and Table 3 summarize the infiltration strategy for SOCs in this section, categorized by metal nanoparticles, perovskites, and other materials.

### 2.1. Metal Infiltration

Among the various methodologies for enhancing electrode performance in solid oxide cells (SOCs), the infiltration of metal nanoparticles into electrode structures emerges as a particularly promising approach. By leveraging the high electrocatalytic activity and extensive surface area of active metal nanoparticles, this technique significantly improves electrode functionality. Specifically, the infiltration of precious metals such as platinum (Pt), palladium (Pd), and silver (Ag) into electrodes has demonstrated substantial improvements in catalytic activity by facilitating superior reaction kinetics and increasing the active sites, thereby enhancing overall electrochemical performance [41,42]. Furthermore, this method allows for the precise and uniform distribution of nanoparticles within the electrode microstructure, maximizing catalyst efficiency while ensuring long-term stability and sustained activity under operational conditions. Beyond precious metals, selected research also investigates the potential of non-precious metals, broadening the scope of material options for cost-effective and high-performance electrode design (Table 1).

Hydrogenated water electrolysis is one of the most prevalent applications of solid oxide cells (SOCs). Orera et al. [43] investigated the infiltration of (Ce/Pr/Mn)O_x_ into LSM-YSZ oxygen electrodes, identifying PrO_x_ as the most effective modifier. This resulted in remarkably low polarization resistances of 0.068 Ω·cm^2^ at 700 °C and 0.018 Ω·cm^2^ at 850 °C. Owing to the high complexity of solid oxide cells (SOCs), the ohmic resistance (Ro) and polarization resistance (Rp) presented in a single-cell impedance process are not fully distinguishable, as their contributions to the overall impedance may overlap. To elucidate the electrode reaction mechanisms in SOCs, the deconvolution distribution of relaxation time (DRT) has been widely adopted in SOC studies [44]. This method enables the interpretation of electrochemical impedance spectroscopy (EIS) data or assists in equivalent circuit model (ECM) design without relying on a priori assumptions. Moreover, high-resolution DRT studies have proven more effective in separating overlapping EIS experimental data [45,46]. Orera et al. analyzed EIS data using both DRT and ECM approaches. The resistance values and peak frequencies obtained from the two fitting methods were found to be largely consistent. However, compared with ECM, DRT analysis can more clearly identify the first relaxation peak, rendering its fitting results more reliable.

**Figure 4:**
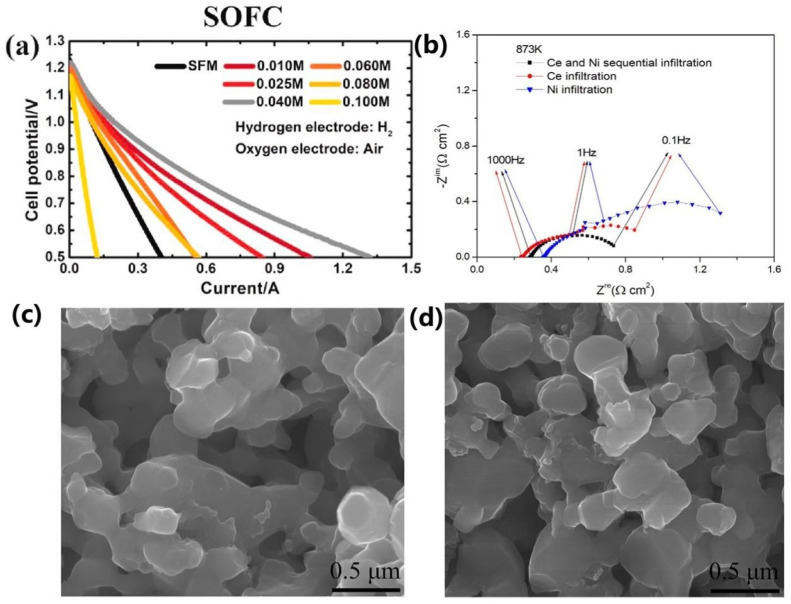
(**a**) I−V curves of cells infiltrated with H_2_PtCl_6_ solutions at different concentrations, measured under (**a**) SOFC mode [47]. Reproduced with permission from [47]. (**b**) Impedance plots of the Ce and Ni sequentially infiltrated NiO−YSZ cell before and after 100 cycles of SOC measurement under open−circuit conditions. Fuel: 17% H_2_O, 5% H_2_, 58% Ar; oxidant: air [48]. Reproduced with permission from [48]. (**c**,**d**) Microstructure of the STFC electrode infiltrated for four cycles, before (**c**) and after (**d**) aging without current [55]. Reproduced with permission from [55].

Among noble metals, platinum has been extensively studied as a catalyst due to its optimal corrosion resistance and high-temperature stability. Lu et al. [47] developed Pt-SFM-SDC electrodes through single-cycle H_2_PtCl_6_ infiltration to optimize performance. These electrodes demonstrated enhanced SOFC performance, as shown in Figure 4a. Similarly, Tatsumi Ishihara et al. [48] investigated the sequential co-diffusion of Ce and Ni on a NiO-YSZ substrate for water hydrogenation electrolysis. After 100 cycles at ±0.1 A·cm^−2^, the OCV remained stable. While simple Ce infiltration showed slight end-potential decreases in SOFC mode with cycling, the sequential infiltration of Ce and Ni improved both performance and stability during SOFC/SOEC cycling, as shown in Figure 4b. The sequential infiltration of Ce and Ni improves the performance and stability and slows down the performance decay due to agglomeration, which is attributed to the inhibition of nanoparticle migration by the NiO-YSZ porous matrix framework and the anchoring effect of the large number of oxygen vacancies.

The co-electrolysis of CO_2_/steam mixtures has emerged as a promising strategy to address CO_2_ emissions and contribute to climate change mitigation. In this context, Hyeon-Ye Jeong et al. [49] introduced a novel approach for optimizing syngas production via high-temperature CO_2_/steam co-electrolysis. Similarly, Tan et al. [50] focused on PdO and ZrO_2_, using them as a catalyst and stabilizer, respectively, to enhance the LSM-YSZ electrode. The co-infiltration method successfully incorporated PdO/ZrO_2_ nanoparticles into the composite electrode, resulting in a significant improvement in electrocatalytic activity. In both SOFC and SOEC modes, this composite electrode demonstrated superior performance, achieving a peak power density of 1.114 W·cm^−2^ and an extraordinary current density of 2.322 A·cm^−2^ under optimized conditions. By comparing the before and after tests, it was found that the PdO/ZrO_2_ nanoparticles grew significantly after the electrolysis test, with the particle size increasing from 35 nm to about 48 nm. From this, it can be inferred that the rapid performance decay in the initial stage is mainly attributed to the coarsening and agglomeration phenomenon of the nanoparticles. Despite the increase in the size of PdO particles after the electrolysis test, the particles stopped continuing to grow after a certain period of continuous operation. The long-term stability during water electrolysis further underscores the viability of this composite for reversible solid oxide electrolysis cells (RSOCs).

The beneficial effects of Pr on both electronic and ionic conductivity in CeO_2_ and super-stoichiometric Ruddlesden–Popper-type (LnNiO_4+δ_-based) structures have been well-documented in the literature [51]. Particularly, Pr6O11 has recently garnered attention for its characteristics as an oxygen electrode material [52]. Khoshkalam et al. [53] infiltrated Pr_6_O_11_ into high-performing submicron LSCF/CGO and low-performing LNF/CGO electrodes, demonstrating a substantial reduction in polarization resistance by a factor of three at temperatures ranging from 600 to 650 °C. Notably, the Pr_6_O_11_-infiltrated LNF/CGO electrodes exhibited performance comparable to, or even surpassing, that of LSCF/CGO electrodes, with remarkably low polarization resistances (0.074 ± 0.002 Ω·cm^2^ at 600 °C and 0.146 ± 0.002 Ω·cm^2^ at 550 °C). The Pr_6_O_11_ infiltration also enhanced durability, showing minimal degradation during a 200 h test at 650 °C (a rate of approximately 17 mΩ.cm^2^/khr (29%/khr)), highlighting its potential for medium-temperature SOFC/SOEC applications. Furthermore, Xu et al. [54] prepared Ni-SFM electrodes by infiltrating Ni in the SFM skeleton.

Wang et al. [56] and Ye et al. [57] both investigated the enhancement in solid oxide electrolysis cells (SOECs) and proton-conducting electrolysis cells (PCECs) through the infiltration of metal nanoparticles into anode materials. Wang et al. [56] demonstrated that nickel (Ni) infiltration into Sr_2_Fe_1.5_Mo_0.5_O_6-δ_ (SFM) and Ce_0.8_Sm_0.2_O_1.9_ (SDC) anodes significantly improved electrochemical performance by increasing current density and reducing applied voltage, facilitated by enhanced methane oxidation. Similarly, Ye et al. [57] focused on the selective infiltration of CeO_2_ onto Ni-BZCYYb electrodes, which improved methane (CH_4_) selectivity and accelerated hydrogenation by enhancing CO_2_ and CO adsorption and proton transfer. Additionally, the infiltration of 1.5 wt% Ru into SFM-SDC further promoted the methane-assisted SOEC process [58]. This body of work underscores the transformative potential of metal nanoparticle infiltration as a cutting-edge methodology in advancing SOC technology.

**Table 1:** Properties of metal nanoparticle-infiltrated materials.

Infiltrated Material	Cell Configuration Anode|Electrolyte| Cathode	T [°C]	Gas Composition	Potential [V]	Current Density [A cm^−2^]	Ref.
(Ce/Pr/Mn)O_x_	LSM/YSZ|YSZ LSM/YSZ	800	H_2_/H_2_O (50:50)	0.7	1.3	[43]
Pt	Ni-YSZ|YSZ|SFM-SDC	800	Oxygen electrode: air Hydrogen electrode: 75% H_2_O-25% H_2_	1.5	3.28	[47]
Ce/Ni	SSC|LSGM|NiO-YSZ	600	Oxygen electrode: air Hydrogen electrode: 17% H_2_O-25% H_2_-58% Ar	~0.9	1.2	[48]
Fe	Ni-YSZ|YSZ|LSCF-GDC	800	H_2_O/CO_2_/H_2_	~1.05	0.4	[49]
PdO	LSM-YSZ|YSZ|Ni-YSZ	750	H_2_O (90 vol%)	2.0	2.322	[50]
Pr_6_O_11_	LSCF|CGO|LSCF	650	/	/	/	[53]
Ni	SFM|LSGM|LSCF-GDC	800	H_2_ (3 vol% H_2_O)	~0.6	0.7	[54]
Ni	SFM-SDC|LSGM| SFM-SDC	850	Cathode: H_2_-H_2_O Anode: CH_4_-H_2_O	0.5	1.022	[56]
CeO_2_	PBSCF-BZCYYb|BZCYYb|BZCYYb	550	Fuel electrode: CO_2_-Ar Oxygen electrode: Ar with 30% H_2_O	1.5	1.12	[57]
Ru	SFM-SDC|LSGM|SFM-SDC	850	Cathode: 74% H_2_O-26% H_2_ Anode: 5.8% CH_4_-91.2% N_2_-3% H_2_O	~0.8	1.1	[58]

### 2.2. Perovskite Infiltration

Perovskites are a class of compounds characterized by the ABO_3_ crystal structure, where the A- and B-sites are occupied by transition metal cations. Specifically, the B-site is coordinated with six oxygen atoms and typically hosts smaller transition metal ions, while the A-site, with 12-fold oxygen coordination, accommodates larger rare earth (RE) elements such as those from the La series. The structural role of RE elements in the perovskite framework enhances charge transfer and stability, making them critical for optimizing electrochemical performance [59]. Sr-doped LaMnO_3_ (LSM) is one of the earliest electrode materials applied to solid oxide cells (SOCs), known for its excellent electronic conductivity (exceeding 100 S cm^−1^ at 800 °C). However, LSM must be sufficiently mixed with ion-conducting electrolyte materials, such as yttria-stabilized zirconia (YSZ) or gadolinium-doped ceria (GDC), to form hybrid composites that exhibit both ionic and electronic conductivity. In this configuration, electrochemical reactions occur exclusively at the three-phase boundary (TPB). Beyond LSM, advanced perovskite derivatives, such as lanthanum strontium cobaltite (LSC, La_1-x_Sr_x_CoO_3_) and samarium strontium cobaltite (SSC, Sm_1-x_Sr_x_CoO_3_), have emerged as promising candidates due to their mixed ionic–electronic conductivity (MIEC). These materials are frequently employed to enhance the ionic conductivity and electrocatalytic performance of oxygen electrodes, making them highly suitable for high-performance electrodes in advanced energy conversion technologies, such as H_2_O and/or CO_2_ electrolysis (Table 2).

Zhang et al. [55] modified La_0.8_Sr_0.2_MnO_3_-Zr_0.92_Y_0.16_O_2-δ_ (LSM-YSZ) composite electrodes by infiltrating SrTi_0.3_Fe_0.6_Co_0.1_O_3-δ_ (STFC). This modification significantly enhanced the electrochemical performance, resulting in a power density of 2200 mW/cm^2^ at 800 °C, over 2.5 times higher than that of the untreated electrodes. The infiltration also led to a substantial reduction in polarization resistance (65%) and improved electrolytic performance, achieving a current density of 2000 mA/cm^2^ at an applied voltage of 1.3 V. Furthermore, the infiltrated electrodes demonstrated excellent stability over 800 h of testing, with no signs of delamination, sintering, or elemental segregation, as shown in Figure 4c,d. Similarly, Vibhu et al. [60] infiltrated La_0.6_Sr_0.4_CoO_3-δ_ (LSC) into La_0.6_Sr_0.4_Co_0.2_Fe_0.3_O_3-δ_ (LSCF) electrodes, reducing polarization resistance from 0.038 Ω·cm^2^ to 0.022 Ω·cm^2^ and achieving a maximum electrolytic current density of 2 A/cm^2^ at 1.5 V. Stability tests at 800 °C for 408 h confirmed that the infiltrated electrodes exhibited improved durability, with minimal degradation and no delamination, largely attributed to the enlarged active sites and inhibition of Sr polarization.

**Figure 5:**
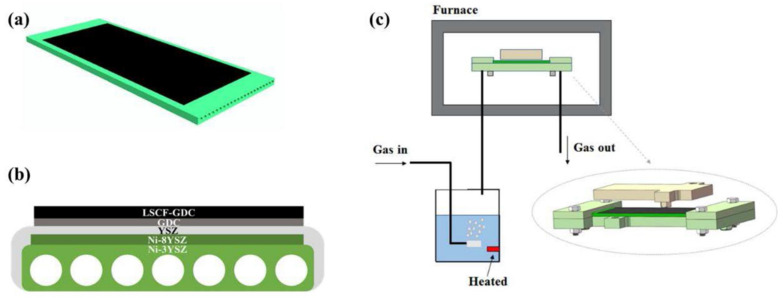
(**a**) Schematic diagram of a flat-tube solid oxide electrolysis cell. (**b**) Cross-sectional structure of the electrolysis cell. (**c**) Schematic representation of the electrolysis test system [63]. Reproduced with permission from [63].

Sun et al. [61] investigated the infiltration of Sr_2_Fe_1.5_Mo_0.5_O_6-δ_ (SFM) into La_0.8_Sr_0.2_Cr_0.5_Fe_0.5_O_3-δ_ (LSCrF)-YSZ electrodes, demonstrating enhanced performance for CO_2_ electrolysis. The modified cells exhibited a current density of 1020 mA/cm^2^ at 800 °C under pure CO_2_ and 1.5 V in electrolysis mode, significantly higher than that of untreated cells (410 mA/cm^2^). On a larger scale, Tong et al. [62] and Song et al. [63] explored the scalability of infiltration techniques in large-area solid oxide cells (SOCs). Tong et al. prepared 4 × 4 cm^2^ (active area) SOCs with La_0.6_Sr_0.4_CoO_3-δ_ (LSC)-infiltrated CGO oxygen electrodes. The results of DRT analysis show that for the cell system with a Ni/YSZ fuel electrode, the resistance of the fuel electrode dominated the overall Rp, while the resistance contribution of the oxygen electrode was relatively small. In fuel cell mode, the cell achieved a power density of 1.34 W·cm^−2^ at 0.6 V and 800 °C. In electrolysis mode, it achieved a current density of 1.37 A·cm^−2^ at 1.3 V and 800 °C, with a vapor utilization of 76%. SEM micrographs of the LSC-CGO oxygen electrodes before and after durability testing did not observe any significant growth in the LSC nanoparticles, demonstrating strong potential for industrial application. Similarly, Song et al. infiltrated Sm_0.5_Sr0_.5_CoO_3-δ_ (SSC) into LSCF-GDC electrodes for large-scale flat-tube solid oxide hydrogen cells, as shown in Figure 5a–c. Their study revealed a significant reduction in degradation rates to 0.031 V/kh over 675 h of operation under electrolysis conditions, improving durability by over 90%. The infiltration suppressed Sr segregation and enhanced the structural stability of the air electrode, underscoring its role in achieving high durability for large-scale hydrogen production. It was shown that the difference in the degree of nickel agglomeration between the infiltration cells and the reference cells was small, and the high magnification SEM photographs of the infiltrated cells after testing showed that the SSC nanoparticles in the air electrodes showed a significant growth trend, which was the main reason for affecting the durability of the cells and leading to the voltage degradation.

**Figure 6:**
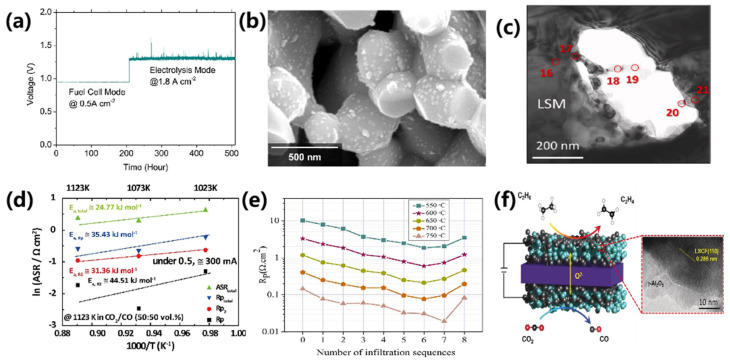
(**a**) Long-term stability of the infiltrated cell measured in fuel cell mode for 200 h and electrolysis mode for 300 h. (**b**) Morphology of the infiltrated nanoparticles after long-term testing [40]. Reproduced with permission from [40]. (**c**) Distribution of the nanoparticles within the functional layer of the LSM/YSZ electrode infiltrated with SrFe_2_O_4-δ_ and operated under SOEC conditions for 900 h [65]. Reproduced with permission from [65]. (**d**) Arrhenius plot of the polarization resistance of the Pd-GDC@LSCM cathode at 0.5 V (loading ~300 mV) over a range of operating temperatures under CO_2_/CO (50:50 vol%) conditions for CO_2_ electrolysis using the SOEC [66]. Reproduced with permission from [66]. (**e**) Magnitude of the electrode polarization resistance of the LSCF electrode for the oxygen reduction reaction (ORR) as a function of the number of LNO infiltration sequences [67]. Reproduced with permission from [67]. (**f**) Coupling of electrochemical ethane and CO_2_ electrolysis into an SOEC, along with a TEM image of the LSCF-SDC anode infiltrated with γ-Al_2_O_3_ [68]. Reproduced with permission from [68].

At the same time, Kyung Joong Yoon et al. [40] significantly enhanced the performance of Sm_0.5_Sr0_.5_CoO_3-δ_ (SSC) nanocatalysis in solid oxide regenerative fuel cells (SORFCs) by employing an innovative infiltration technique based on urea decomposition. This method facilitates precise control over the size, morphology, and spatial distribution of nanoparticles, thereby mitigating common challenges such as high-temperature instability. The optimized process results in an electrode structure that closely aligns with the ideal configuration predicted by modeling studies. These findings highlight the efficacy of infiltration techniques in improving both the performance and durability of solid oxide electrolysis cells, as illustrated in Figure 6a,b. Furthermore, this advancement promotes the scalability of solid oxide cell (SOC) technology for industrial applications. Finally, Lu et al. [64] infiltrated Sm_0.5_Sr0_.5_CoO_3-δ_ (SSC) (SSC) nanoparticles into LSCF-GDC oxygen electrodes, significantly enhancing stability. Over 1000 h at 650 °C, the polarization resistance of non-infiltrated electrodes increased 105-fold, while SSC-infiltrated electrodes showed minimal change, demonstrating the effectiveness of SSC in mitigating degradation.

**Table 2:** Properties of perovskite-infiltrated materials.

Infiltrated Material	Cell Configuration Anode|Electrolyte| Cathode	T [°C]	Gas Composition	Potential [V]	Current Density [A cm^−2^]	Ref.
SrTi_0.3_Fe_0.6_Co_0.1_O_3-δ_(STFC)	LSM-YSZ|YSZ|LSM-YSZ	700	Cathode: air Anode: 97% H_2_ + 3% H_2_O	~0.8	0.5	[55]
La_0.6_Sr_0.4_CoO_3-δ_ (LSC)	LSCF|YSZ|LSCF	800	50%: 50% (H_2_: H_2_O)	1.4	1.75	[60]
Sr_2_Fe_1.5_Mo_0.5_O_6−δ_ (SFM)	LSM-YSZ|YSZ|LSCF-YSZ	800	Fuel electrode: pure CO_2_ Air electrode: air	1.5	1.02	[61]
La_0.6_Sr_0.4_CoO_3-δ_ (LSC)	LSC-CGO|YSZ|NiO/YSZ	750	Fuel electrode: 90% H_2_O-10% H_2_ Air electrode: pure O_2_	1.3	1.07	[62]
Sm_0.5_Sr_0.5_CoO_3-δ_ (SSC)	LSCF-GDC|YSZ|NiO-YSZ	750	76% H_2_O-24% H_2_	1.29	0.761	[63]
Sm_0.5_Sr_0.5_CoO_3-δ_(SSC)	LSCF-GDC|YSZ|NiO-YSZ	750	Fuel electrode: 97% H_2_-3% H_2_O Air electrode: air	1.29	2.1	[40]
Sm_0.5_Sr_0.5_CoO_3-δ_ (SSC)	LSCF-GDC|GDC|LSCF-GDC	650	Air	/	/	[64]

### 2.3. Other Infiltration

In addition to metal nanoparticles and perovskites, gadolinium-doped ceria (GDC) and samarium-doped ceria (SDC), known for their superior ionic conductivity, are effective options for enhancing the performance of solid oxide cells. Furthermore, oxides with Ruddlesden–Popper (R-P) structures (A_2_BO_4_), such as La_2_NiO_4_ (LNO), are extensively utilized in SOCs due to their excellent electron–ion conductivity and stability under both oxidizing and reducing conditions (Table 3).

Brito et al. [69] developed high-performance oxygen electrodes for reversible solid oxide cells (RSOCs) by infiltrating (CeO_2_)_0.8_(Sm_2_O_3_)_0.2_ (SDC) nanoparticles into a La_0.6_Sr_0.4_Co_0.2_Fe_0.8_O_3-δ_ (LSCF)-(CeO_2_)_0.8_(Sm_2_O_3_)_0.2_ (SDC) composite electrode. With a 30 vol% SDC nanoparticle volume fraction, the electrode demonstrated a low overpotential and significant performance improvement. The microstructure of the electrode was observed after a series of polarization tests, and only a small number of SDC nanoparticles were observed to be slightly agglomerated in the SEM images, leaving the microstructure of the electrode virtually unchanged. At 750 °C, the constant overpotential of η = 0.020 V increased the current density by a factor of 4 (from 0.03 to 0.13 A·cm^−2^) compared to non-infiltrated electrodes. Both the overpotential (η) and ohmic resistance (Rₒₕₘ) remained stable during 400 h of electrolysis at 0.5 A·cm^−2^. This infiltration effectively improved both performance and stability under anodic and cathodic reactions, making it a promising approach for RSOCs. Similarly, Fan et al. [65] infiltrated SrFe_2_O_4-δ_ into LSM/YSZ air electrodes, reducing delamination, slowing performance degradation, and extending SOEC life. Over 240 h, the polarization resistance of the infiltrated cell was reduced by nearly two orders of magnitude compared to the baseline cell following SrFe_2_O_4-δ_ infiltration in the air electrode. The SrFe_2_O_4-δ_ nanoparticles maintained their morphology and distribution within the pores for 900 h, as shown in Figure 6c, enhancing ionic conductivity and electrocatalytic activity through continuous cation exchange with the LSM backbone. This surface modification significantly improved ionic conductivity, hydrogen production, and cell stability in SOEC applications.

LSCM is a promising cathode material for high-temperature CO_2_ electrolysis due to its high redox stability and conductivity under reducing conditions. Lee et al. [66] developed Pd-GDC@LSCM by co-infiltrating Pd-GDC nanoparticles, achieving an activation energy for the area-specific resistance of 49.2 kJ·mol^−1^ at open-circuit voltage and 24.77 kJ·mol^−1^ at 0.5 V, as shown in Figure 6d. These values were lower than those reported previously. The Pd-GDC@LSCM material, characterized by micrometer-sized pores and dispersed Pd-GDC nanoparticles, improved CO_2_ and CO gas permeation and extended the three-phase boundary, thereby enhancing electrochemical activity. Similarly, Lv et al. [70] infiltrated GDC nanoparticles into Sr_2_Fe_1.5_Mo_0.5_O_6-δ_ (SFM) to create a GDC-SFM composite oxygen electrode for CO_2_ electrolysis, which significantly improved redox performance. The maximum current density was 446 mA/cm^2^ at 800 °C and 1.6 V with a 12.8% infiltration content, significantly higher than that of the un-infiltrated cell.

LNO is a mixed ionic and electronic conductor characterized by highly mobile per stoichiometric oxygen, which exhibits high oxygen ionic conductivity along with excellent oxygen surface exchange and diffusion coefficients [71,72]. Ghamarinia et al. [67] prepared LNO-LSCF cathodes by infiltrating nanoscale LNO with varying Ruddlesden–Popper (R-P) structure loadings into an LSCF skeleton. DRT analyses showed that the performance enhancement stemmed from the excellent ionic conductivity, bulk oxygen diffusion capability, oxygen surface exchange activity, and nanoscale particle properties of the R-P structured LNO materials. The LSCF cathode with six LNO infiltrations achieved an average 81% reduction across all temperatures, as shown in Figure 6e. It was shown that a negative correlation between temperature and polarization resistance could be observed, as the polarization resistance increased at all temperatures when the infiltration process was conducted more than six times. This phenomenon could be attributed to the agglomeration of nanoparticles at high temperatures caused by excessive infiltration. Such agglomeration altered the morphology of the electrode material, thereby reducing the active sites for the oxygen reduction reaction (ORR). Additionally, the closure of pores within the LSCF skeleton hindered oxygen diffusion. These findings indicate that both infiltration temperature and quantity play crucial roles in determining the performance of the electrode reaction. Similarly, Liu et al. [73] found that twice-infiltrated La_2_NiO_4_ layers on La_0.8_Sr_0.2_Co_0.8_Ni_0.2_O_3-δ_ (LSCN) electrodes reduced degradation and improved oxygen surface exchange and diffusion under SOEC conditions, highlighting the effectiveness of LNO infiltration in enhancing cathode performance.

Song et al. [68] investigated γ-Al_2_O_3_ as an infiltration material to enhance the electrochemical oxidative dehydrogenation (ODE) of ethane in solid oxide electrolysis cells (SOECs), as shown in Figure 6f. Infiltrating γ-Al_2_O_3_ onto La_0.6_Sr_0.4_Co_0.2_Fe_0.8_O_3-δ_ (LSCF) and Sm_0.2_Ce_0.8_O_2-δ_ (SDC) surfaces improved ethylene selectivity to 92.5% and ethane conversion to 29.1% at 600 °C. The interaction between Al_2_O_3_ and the LSCF anode reduced adsorbed oxygen species, thereby enhancing the selectivity towards ethylene over deep oxidation products. Density functional theory (DFT) and in situ X-ray photoelectron spectroscopy (XPS) analyses revealed that Al-O-Fe bond formation altered the anode’s electronic structure, thus enhancing ethane adsorption and conversion efficiency.

In summary, the infiltration process is an efficient technique wherein a precursor solution is introduced into a porous scaffold and thermally treated to form the desired active phase. It is worth mentioning that the most obvious problem with infiltration is that the infiltrated particles tend to agglomerate at high temperatures over time, thus reducing the performance of the SOC. This can be effectively addressed by optimizing the infiltration process parameters to control the infiltration temperature and concentration and by enhancing the Metal–Support Interaction with the use of porous and high-specific-surface-area carriers. In conclusion, infiltration improves the performance and adaptability of the electrodes, providing good phase control, better material distribution, and enhanced electrochemical performance compared to conventional fabrication methods.

**Table 3:** Properties of other infiltrated materials.

Infiltrated Material	Cell Configuration Anode|Electrolyte| Cathode	T [°C]	Gas Composition	Potential [V]	Current Density [A cm^−2^]	Ref.
SDC	LSCF-SDC|YSZ|LSCF-SDC	800	Cathode and anode: pure O_2_	0.03	0.5	[69]
SrFe_2_O_4_-_δ_	LSM/YSZ|YSZ|Ni-YSZ	800	SOEC: Cathode: 60% steam Anode: air SOFC: 100% H_2_	~1.2	2.0	[65]
Pd-GDC	LSCF|YSZ|LSCM	850	Cathode: 50% CO_2_/CO Anode: air	1.5	0.364	[66]
Gd_0.2_Ce_0.8_O_1.9_ (GDC)	LSM-YSZ|YSZ|SFM	800	95% CO_2_/5% N_2_	1.6	0.446	[70]
La_2_NiO_4_	LSCF|YSZ|LSCF	650	O_2_	/	/	[67]
La_2_NiO_4+δ_ (LNO)	LSCN|GDC|LSCN	750	air	0.104	0.5	[73]
γ-Al₂O₃	LSCF-SDC|LSGM|LSCF-SDC	600	Cathode: 95% CO_2_ + 5% N_2_ Anode: 36% C_2_H_6_ + 5% N_2_ + 59% Ar	/	/	[68]

## 3. Exsolution

**Figure 7:**
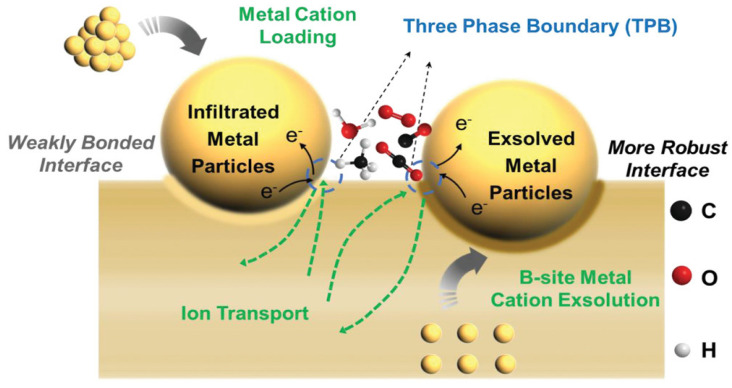
Comparison of oxide-supported metal particle catalysts prepared via infiltration and the in situ exsolution strategy. Ref. [6]. Reproduced with permission from [6].

Metal nanoparticles can exsolve from the crystal lattice to the surface of specific oxide matrices following thermal annealing under reducing conditions or the application of appropriate electric potentials [33]. As depicted in Figure 7, these exsolved metal particles are typically embedded within oxide matrices, forming stable heterointerfaces. These interfaces facilitate robust interactions between metal particles and oxide matrices, thereby enhancing electron–ion transport across the metal–oxide interface. This interaction promotes the formation of abundant triple-phase boundaries while mitigating the agglomeration or growth of metal particles [74,75,76]. Moreover, compared to exogenous metal particles, exsolved nanoparticles exhibit a more uniform distribution on oxide surfaces and smaller particle sizes under equivalent metal loading conditions. In this section, we systematically review the exsolution strategies categorized by various single-metal (Section 3.1) and alloy exsolution methods (Section 3.2). These strategies are also summarized in Table 4, Table 5, Table 6, Table 7, Table 8, Table 9 and Table 10.

### 3.1. Exsolution of Single-Metal Nanoparticles

Single-metal nanoparticles typically exhibit high surface energy and abundant active sites. During electrolysis, the exsolution of metal nanoparticles on the perovskite surface enhances the catalytic activity of the electrodes [77], thereby improving the reaction rate and increasing the electrode surface area and porosity. This facilitates the diffusion and transport of reactive gases. This section reviews four common single-metal nanoparticles—Co, Cu, Ni, and Fe—on the perovskite surface, along with various control strategies to promote nanoparticle exsolution and enhance solid oxide cell performance.

#### 3.1.1. Co Nanoparticles

Cobalt exhibits superior chemical stability and electronic conductivity at high temperatures. The “crater” structure formed during the exsolution of cobalt nanoparticles anchors them firmly on the perovskite surface, effectively preventing nanoparticle agglomeration and loss. This structural feature endows the material with excellent catalytic performance in solid oxide cell reactions, thereby significantly enhancing the overall performance of the cell.

Li et al. [78] investigated the stability of perovskite cathode materials, including La_0.5_Sr_0.5_Fe_0.8_Co_0.2_O_3-δ_(LSFC82), La_0.5_Sr_0.5_Fe_0.7_Co_0.2_Nb_0.1_O_3-δ_(LSFCN721), and La_0.5_Sr_0.5_Fe_0.8_Co_0.1_ Nb_0.1_O_3-δ_ (LSFCN811), for CO_2_ electrolysis in solid oxide electrolysis cells (SOECs). Under a 95%CO_2_/5%N_2_ atmosphere, Co nanoparticles exsolved on LSFC82, whereas Nb-doped materials (LSFCN721 and LSFCN811) exhibited limited exsolution, indicating that Nb inhibits this process. LSFC82-SDC achieved a higher current density of 1.8 A·cm^−2^ at 800 °C and 1.6 V compared to 1.56 A·cm^−2^ for Nb-doped materials, demonstrating the performance benefits of Co nanoparticle exsolution. Lu et al. [79] developed Ce-LCCo, a composite cathode doped with CeO_2_ and Co on a chromite framework, emphasizing the role of oxygen storage in CO_2_ electrolysis. After 30 h of timed current testing, the Ce-LCCo cathode surface precipitated visible particles (30 nm in size), which gradually grew to about 100 nm in diameter. More importantly, the density of such particles increased significantly with the durability test. It is noteworthy that the size of Co particles after the 30 h durability test was much smaller than that of the 300 h test sample, suggesting that durability testing under cathodic current leads to partial pulverization of the cathode material. The reduction of Ce-LCCo at 800 °C in a 5% H_2_ atmosphere led to the exsolution of Co nanoparticles and CeO_2_, thereby enhancing catalytic activity. This highlights the advantages of nanoparticle exsolution and oxygen storage capacity.

**Figure 8:**
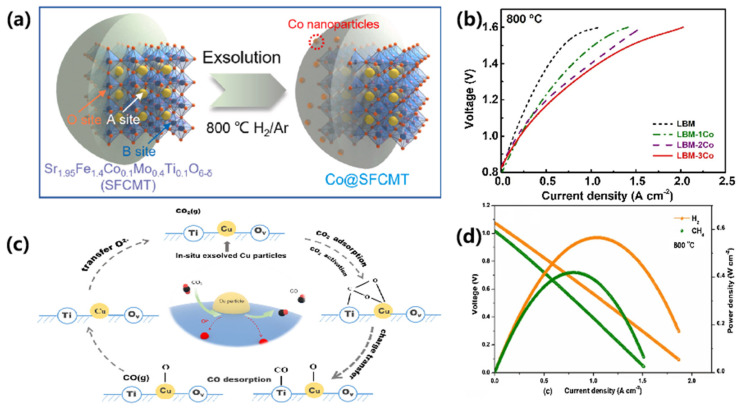
(**a**) Schematic illustration of the in situ exsolution mechanism for Sr_1.9_Fe_1.3_Cu_0.2_Mo_0.4_Ti_0.1_O_6-δ_ (SFCMT) under a reducing atmosphere [80]. Reproduced with permission from [80]. (**b**) H_2_O-CO_2_ co-electrolysis performance curves of cells with various cathodes at 800 °C [82]. Reproduced with permission from [82]. (**c**) Schematic diagram of the CO_2_ reduction process mechanism [83]. Reproduced with permission from [83]. (**d**) Cell voltages and power densities as functions of current density for LSFCu symmetrical electrodes measured in fuel cell (FC) mode using H_2_ and CH_4_ as fuels [84]. Reproduced with permission from [84].

Gao’s group [80] designed Sr_1.95_Fe_1.4_Co_0.1_Mo_0.4_Ti_0.1_O_6-δ_ (SFCMT), where the partial substitution of Co for Fe promoted Co nanoparticle exsolution via the non-stoichiometric modulation of Sr sites, as shown in Figure 8a. The cathode achieved a current density of 2.57 A·cm^−2^ at 1.8 V and 800 °C, demonstrating stability over 200 h at 1.5 V and 750 °C. Similarly, Yang et al. [81] utilized a double perovskite, Sr_2_Fe_1.3_Co_0.2_Mo_0.5_O_6-δ_ (SFCM), where Co nanoparticle exsolution during electrolysis was confirmed through XRD, XPS, TEM, and SEM analyses. This resulted in reduced polarization resistance (0.24 Ω·cm^2^ under open-circuit voltage in a 50% H_2_O-50% H_2_ atmosphere) and enhanced electrochemical performance. Notably, SFCM demonstrated remarkable durability with negligible degradation during 20 h of CO_2_ electrolysis at 0.4 A·cm^−2^ and 50 h of CO_2_/H_2_O co-electrolysis at 1 A·cm^−2^. These results establish SFCMT and SFCM as promising materials for future SOECs.

Gan et al. [82] synthesized La_0.5_Ba_0.5_Mn_1-x_Co_x_O_3-δ_ (x < 0.4) with a perovskite structure as a cathode material for SOECs and conducted in situ studies on the exsolution of Co from the perovskite under reducing atmospheres. The exsolution of Co enhanced the electrical conductivity of La_0.5_Ba_0.5_MnO_3-δ_ in reducing environments and facilitated oxygen exchange reactions on the cathode surface, demonstrating high activity for both H_2_O and CO_2_ electrolysis, as shown in Figure 8b. The cathode also successfully enabled the co-electrolysis of H_2_O and CO_2_, with the CO_2_ content in the reactant gas influencing the CO/H_2_ ratio in the production.

**Table 4:** Properties of Co exsolution materials.

Exsolved Metal	Electrode Composition	T [°C]	Gas Composition	Potential [V]	Current Density [A cm^−2^]	Ref.
Co	La_0.5_Sr_0.5_Fe_0.8_Co_0.2_O_3-δ_-SDC	800	5% N_2_/95% CO_2_	1.6	1.80	[78]
CeO_2_/Co	La_0.7_Ca_0.3_CrO_3_	800	Pure CO_2_	1.3	1.05	[79]
Co	Sr_1.95_Fe_1.4_Co_0.1_Mo_0.4_Ti_0.1_O_6-δ_ (SFCMT)	800	Pure CO_2_	1.8	2.57	[80]
Co	Sr_2_Fe_1.3_Co_0.2_Mo_0.5_O_6-δ_	850	50% CO_2_–50% CO	1.4	2.12	[81]
Co	La_0.5_Ba_0.5_Mn_0.9_Co_0.1_O_3-δ_	800	Cathode: 10% H_2_O-90% H_2_ or 4% CO-96% CO_2_ Anode: air	1.3	1.01	[82]

#### 3.1.2. Cu Nanoparticles

High-efficiency carbon dioxide electrolysis facilitated by Cu nanoparticle exsolution was demonstrated by Yang et al. [83] and Cui [85] et al. Yang et al. [83] investigated the in situ exsolution of Cu nanoparticles on a perovskite surface through the synthesis of an A-site-deficient (La_0.2_Sr_0.8_)_0.9_Ti_0.5_Mn_0.4_Cu_0.1_O_3-δ_ (LSTMC) material, which was reduced in an H₂ atmosphere at 800 °C. The CO_2_ reduction mechanism is illustrated in Figure 8c. The exsolved Cu nanoparticles, integrated with the perovskite matrix, formed a heterogeneous structure that significantly increased the density of active sites, thereby enhancing catalytic performance. DRT analyses showed that the in situ exsolution of Cu nanoparticles promoted both CO_2_ adsorption and electrochemical reduction. As a cathode, the reduced LSTMC achieved a current density of 2.82 A·cm^−2^ at 1.8 V and 800 °C, approximately 2.5 times higher than that of the un-doped LSTM counterpart. Building on these strategies, Cui et al. [85] synthesized Sr_1.9_Fe_1.3_Cu_0.2_Mo_0.4_Ti_0.1_O_6-δ_ (SFCMT) with A-site deficiencies to promote uniform exsolution of Cu nanoparticles on the perovskite surface. This process enhanced oxygen vacancy concentrations, providing additional active sites and further improving electrocatalytic activity. The SFCMT cathode exhibited an outstanding current density of 3.21 A·cm^−2^ at 1.8 V and 800 °C, coupled with a low polarization resistance of 0.2 Ω·cm^2^, demonstrating its exceptional catalytic efficiency and advanced material design.

Seungyeon Jo et al. [86] synthesized Cu nanoparticles (NPs) via in situ exsolution from La_0.43_Sr_0.37_Cu_0.12_Ti_0.88_O_3₋δ_ (LSCuT) perovskite, demonstrating superior thermal stability compared to infiltration methods. The exsolved Cu NPs exhibited excellent stability at 900 °C for 150 h in a 3% H_2_O/H_2_ atmosphere, and electrochemical reduction (ER) facilitated rapid NP growth. A single solid oxide fuel cell (SOFC) with an LSCuT anode achieved a peak power density of 1.38 W·cm^−2^ at 900 °C. These findings indicate that exsolved Cu NPs enhance both performance and stability, offering significant potential for broader applications in SOFCs and SOECs.

Yang et al. [87] investigated the exsolution behavior of metal nanoparticles in solid oxide cells by synthesizing a series of Sr_x_Ti_0.7_Cu_0.2_Mo_0.1_O_3₋δ_ (SₓTCM, x = 1, 0.975, 0.95) perovskites, employing stoichiometric modulation of the A-site to enhance the solvation of B-site Cu metal ions. The introduction of A-site defects was shown to increase the concentration of oxygen vacancies, thereby enhancing oxygen ion transport capacity and reaction kinetics. Among the three levels of Sr incorporation, the Sr_0.975_TCM cathode exhibited superior performance based on extensive characterization tests. The cell achieved a current density of 2.16 A·cm^−2^ at 800 °C with an applied voltage of 1.8 V, while maintaining Faraday efficiency close to 100%. These findings highlight the significant enhancement in electrochemical performance in both solid oxide fuel cell (SOFC) and solid oxide electrolysis cell (SOEC) modes through defect regulation in the S_x_TCM structure.

Fu et al. [84] investigated the leaching mechanism of Cu nanoparticles in (La_0.9_Sr_0.1_)Fe_0.9_Cu_0.1_O_4_ (LSFCu) with A-site defects, demonstrating that LSFCu exhibits high electrocatalytic activity for both the oxygen reduction reaction (ORR) and fuel oxidation reaction (FOR), thereby serving simultaneously as a fuel electrode and an oxygen electrode. As illustrated in Figure 8d, in fuel cell (FC) mode, when using humidified H₂ and CH₄ as fuels, the peak power densities at 800 °C were 573 mW·cm^−2^ and 396 mW·cm^−2^, respectively. Furthermore, in electrolysis cell (EC) mode, a very high current density of 1.02 A·cm^−2^ was achieved at 1.2 V. Their work provides a novel strategy for utilizing a single electrode material in solid oxide cells (SOCs) to simultaneously convert various types of energy, enabling high-activity fuel and oxygen electrodes.

**Table 5:** Properties of Cu exsolution materials.

Exsolved Metal	Electrode Composition	T [°C]	Gas Composition	Potential [V]	Current Density [A cm^−2^]	Ref.
Cu	(La_0.2_Sr_0.8_)_0.9_Ti_0.5_Mn_0.4_Cu_0.1_O_3−δ_	800	Pure CO_2_	1.8	2.82	[83]
Cu	Sr_1.9_Fe_1.3_Cu_0.2_Mo_0.4_Ti_0.1_O_6-δ_	800	CO_2_	1.8	3.21	[85]
Cu	La_0.43_Sr_0.37_Cu_0.12_Ti_0.88_O_3-δ_	900	3% H_2_O/H_2_	~0.7	1.5	[86]
Cu	Sr_x_Ti_0.7_Cu_0.2_Mo_0.1_O_3-δ_	800	SOEC: pure CO_2_ SOFC: pure H_2_	1.8	2.16	[87]
Cu	(LaSr)_0.9_Fe_0.9_Cu_0.1_O_4_	800	Fuel cell: H_2_ and CH_4_ Electrolysis cell: 53.2% H_2_O/46.8% N_2_	~1.2	1.02	[84]

#### 3.1.3. Ni Nanoparticles

Nickel-based catalysts are extensively utilized in energy conversion and storage devices, particularly in solid oxide fuel cells (SOFCs) and solid oxide electrolysis cells (SOECs), owing to their numerous advantages. These catalysts exhibit high catalytic activity in critical reactions, such as CO_2_ electrolysis, CO_2_/H_2_O co-electrolysis, and ethane electrochemical oxidation dehydrogenation (EODHE). Nickel-based catalysts possess excellent thermal stability and strong electrical conductivity [88]. Simultaneously, Ni-based catalysts in electrode materials can enhance electrochemical activity and long-term stability [89]. This section of this review examines the exsolution behavior of Ni nanoparticles in solid oxide cells, addressing key challenges and recent advancements in this field.

**Figure 9:**
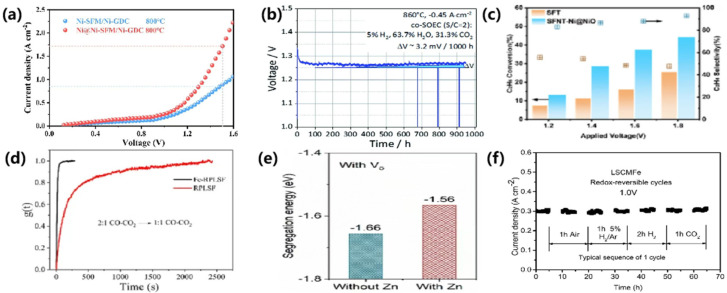
(**a**) I−V curves of Ni-SFM/Ni-GDC and Ni@Ni−SFM/Ni−GDC-based SOECs [90]. Reproduced with permission from [90]. (**b**) Voltage vs. time during long-term co-electrolysis (co-EC) with a fuel gas composition of 5% H_2_, 63.7% H_2_O, and 31.3% CO_2_ at 860 °C and 0.46 A·cm^−2^ for the L65SCrN fuel electrode [93]. Reproduced with permission from [93]. (**c**) C_2_H_6_ conversion and C_2_H_4_ selectivity at 800 °C [94]. Reproduced with permission from [94]. (**d**) ECR curves of Fe-RPLSF and RPLSF bars at 850 °C [95]. Reproduced with permission from [95]. (**e**) Comparison of the segregation energy of Fe before and after Zn doping [96]. Reproduced with permission from [96]. (**f**) Redox-cycling performance of the single cell with LSCMFe-SDC at 850 °C [97]. Reproduced with permission from [97].

Hu et al. [90] developed a self-assembled dual exsolution cathode strategy to enhance CO_2_ electrolysis performance. By doping Ni into SrFe_0.7_Mo_0.3_O_3-δ_ (SFM) and Gd_0.1_Ce_0._9O_1.95_ (GDC) to form a Ni-SFM/Ni-GDC cathode, Ni nanoparticles were precipitated in situ on the surface via reduction at 800 °C under a hydrogen atmosphere. This approach resulted in a cathode with excellent CO selectivity and durability, significantly improving the CO_2_ reduction reaction kinetics. The cell achieved a current density of 1.72 A·cm^−2^ at 800 °C and 1.5 V, as shown in Figure 9a. Similarly, Zhang et al. [91] investigated the effect of Mn doping on La_0.3_Ca_0.6_Ni_0.05_Ti_0.95_O_3₋γ_ perovskite materials for CO_2_ electrolysis. Mn, as a B-site dopant, enhanced the reducibility of Ni, facilitating in situ exsolution of Ni nanoparticles under a reducing atmosphere. The La_0.3_Ca_0.6_Ni_0.05_Ti_0.95_O_3₋γ_ cathode achieved a maximum current density of 2.89 A·cm^−2^ at 1.8 V and 900 °C, with exsolution Ni nanoparticles significantly improving catalytic performance for CO_2_reduction. Furthermore, Liu et al. [92] proposed a layered perovskite material, (La_4_Srn)_0.9_Ti_0.9n_Ni_0.1n_O_3n+2_ (*n* = 5, 8, 12), as a cathode for CO_2_ electrolysis. A-site defects and Ni doping in the B-site facilitated the exsolution of Ni, promoting efficient CO_2_ electrolysis. The synergistic effect of abundant oxygen vacancies and Ni nanoparticles on the perovskite surface enhanced catalytic performance, with LSTN₈ reaching a current density of 1.50 A·cm^−2^ at 800 °C and 2.0 V, highlighting its potential for efficient CO_2_ reduction.

Rémí Costa’s research team [93] introduced a novel La_0.65_Sr_0.30_Cr_0.8_5Ni_0.15_O_3₋δ_ (L65SCrN) fuel electrode modified in situ with Ni nanoparticles, which demonstrated excellent performance under co-electrolysis conditions. The electrode achieved a total area-specific resistance of 0.676 Ω·cm^2^ at a current density of 0.3 A·cm^−2^. During reversible solid oxide cell (rSOC) operation at 860 °C with a H_2_O/H_2_ ratio of 1, and under co-electrolysis at 860 °C with a H₂O/CO₂ ratio of 2 at 0.45 A·cm^−2^, voltage degradation was minimal (less than 3.5 mV/1000 h), with stable operation maintained for up to 950 h, as shown in Figure 9b. These results highlight the potential of the L65SCrN electrode for SOC applications, further enhanced by the exsolution of Ni nanoparticles. However, the size and stability of Ni nanoparticles, influenced by factors such as temperature and exposure duration, are critical for optimizing long-term performance. Further investigation into nanoparticle exsolution is essential to refine electrode materials and improve their behavior under SOC operating conditions.

Ethane electrochemical oxidation dehydrogenation (EODHE) in ceramic reactors is emerging as a promising method for efficient energy conversion and the synthesis of high-value products. A key study by Zhang et al. [94] explored a Ni-NiO_x_ core–shell structure, specifically, the Ni@NiO configuration, supported on a Sr_3_Fe_1.4_Ni_0.1_Ti_0.5_O_7₋δ_ porous bilayer perovskite oxide. This material was tested as an anode electrode for EODHE in solid oxide fuel cells (SOFCs). The study demonstrated that the nanoscale interface between Ni@NiO nanoparticles and the perovskite oxide support improved the reaction kinetics for the adsorption, exsolution, and oxidation of ethane (C_2_H_6_). The Ni@NiO interface efficiently transferred active sites from the oxide support to the nanoparticle surface, thereby boosting catalytic performance. As shown in Figure 9c, under conditions of 1.8 V and 800 °C, the electrode achieved an impressive ethane conversion rate of 44.3% with an ethylene selectivity of 92.7%. These results highlight the potential of this electrochemical dehydrogenation process and material design for advancing clean energy technologies.

**Table 6:** Properties of nickel exsolution materials.

Exsolved Metal	Electrode Composition	T [°C]	Gas Composition	Potential [V]	Current Density [A cm^−2^]	Ref.
Ni	Ni@Ni-SFM/NiGDC	800	Pure CO_2_	1.5	1.72	[90]
Ni	La_0.3_Ca_0.6_Ni_0.05_Mn_x_Ti_0.95-x_O_3-γ_	900	77.6% CO_2_-19.4% H_2_-3% H_2_O	1.8	2.89	[91]
Ni	(La_4_Srn_4_)_0.9_Ti_0.9n_Ni_0.1n_O_3n+2_ (n = 5, 8, and 12)	800	85% CO_2_-CO	2.0	1.5	[92]
Ni	La_0.70_Sr_0.3_Cr_0.85_Ni_0.15_O_3−δ_ (L_70_SCrN)	860	H_2_-H_2_O-CO_2_	1.4	~1.0	[93]
Ni	Sr_3_Fe_1.4_Ni_0.1_Ti_0.5_O_7−δ_	800	Anode: 50% C_2_H_6_ + 50% Ar Cathode: CO_2_	1.8	~1.3	[94]

#### 3.1.4. Fe Nanoparticles

Iron-based materials are considered promising alternatives to precious metals such as Ag and Pd, owing to their favorable electrochemical properties, high thermal stability, and lower cost [98]. In particular, nanostructured iron-based materials can enhance surface area and active sites, thereby improving catalytic performance and optimizing the cycling stability of cells [99]. The in situ exsolution of Fe has garnered significant attention in the field of metal exsolution due to its availability, cost-effectiveness, and high catalytic activity. This section of this review provides an overview of current research on the exsolution behavior of iron nanoparticles in solid oxide cells.

The exsolution of Fe nanoparticles in CO_2_ electrolysis reactions has been extensively investigated, with a focus on enhancing the performance and stability of cathode materials. Liu et al. [95] developed a highly active and low-cost cathode material (Fe-RPLSF), where Fe nanoparticles were reduced in situ on the perovskite surface under a dry hydrogen atmosphere at 850 °C. This exsolution of Fe nanoparticles not only enhanced ionic conductivity but also improved the CO_2_ dissociative adsorption process. As shown in Figure 9d, the reduction in relaxation time indicates faster CO_2_ reduction on Fe-RPLSF oxides. The resulting electrolytic cell, employing Fe-RPLSF as the cathode material, achieved an impressive current density of 1920 mA·cm^−2^ at 850 °C and 1.5 V, demonstrating the potential of Fe nanoparticle-based materials in CO_2_ electrolysis. In a similar vein, Tan et al. [100] synthesized a double perovskite material, (Pr_0.5_Ba_0.5_)_1.8_Fe_1.8_Mn_0.2_O_5₋δ_ (PBFM), where Fe nanoparticles were solvated and promoted by A-site defects. The phase reconstruction and solvation of Fe nanoparticles increased the number of oxygen vacancies and reactive sites, leading to improved CO_2_ electrolysis performance. The current density for this material reached 1.6 A·cm^−2^ at 850 °C and 1.8 V, maintaining excellent stability during a 100 h test. Delamination and carbon deposition could not be observed after long-term testing, implying excellent cell stability.

To further enhance catalytic performance, Liu et al. [96] proposed a passivation reconstruction strategy involving the partial substitution of Fe with Zn in the perovskite matrix (SFM), which led to an increase in Fe-segregation energies, as shown in Figure 9e. This strategy increased the concentration of oxygen vacancies, thereby diminishing the in situ exsolution of Fe nanoparticles and enhancing catalytic activity. The optimal doping of Zn resulted in significant performance improvements, achieving a maximum current density of 2.74 A·cm^−2^ at 850 °C and 1.6 V. This enhancement compared favorably with the SFM substrate material, showcasing the potential of doped perovskite materials in CO_2_ electrolysis. Gao et al. [101] further addressed the interfacial stability and carbon resistance of metal oxides by designing a series of B-site-doped SrFe_1.5+x_M (SF_1.5+x_ M) as symmetric electrodes. Their voltage-driven reduction process, requiring only 180 s, effectively anchored Fe nanoparticles on the perovskite surface, preventing sintering and carbon accumulation. This approach facilitated CO_2_ adsorption and enhanced ionic electron transport, achieving a polarization impedance of 0.155 Ω·cm^2^ and a CO yield of 4.0 mL·min^−1^·cm^−2^ at 850 °C and 1.6 V.

Choi et al. [102] investigated the exsolution of Fe nanoparticles on the surface of La_1.2_Sr_0.8_Mn_0.4_Fe_0.6_O_4_(LSMF) perovskite under reducing conditions, leading to a significant increase in oxygen vacancies within the perovskite matrix. This exsolution process notably enhanced the electrocatalytic performance. After reduction, the system achieved a current density of 2.04 A·cm^−2^ at 850 °C and 1.5 V, with a Faraday efficiency approaching 100%. Similarly, Jiang et al. [103] explored the use of deironized cerium dioxide (SDC) as an electrolytic CO_2_ cathode material, finding that doping Sm into CeO_2_ helped prevent the formation of SrCO_3_ during the reaction. Moreover, the incorporation of 10% Fe into the SDC via B-site doping, followed by reduction under a 50% CO/CO_2_ atmosphere, led to the formation of dispersed Fe nanoparticles. This approach significantly enhanced the catalytic activity for CO_2_ electrolysis, further highlighting the role of Fe nanoparticles in improving electrocatalytic performance under reducing conditions.

A study by Dong et al. [97] highlighted the potential of in situ exsolved Fe nanoparticles on perovskite supports to enhance the electrocatalytic reforming of CO_2_ and CH_4_ in solid oxide electrolysis cells (SOECs). By doping Fe into the perovskite lattice of La_0.75_Sr_0.25_Cr_0.5_Mn_0.5_O_3₋δ_ (LSCM), the innovative use of active metal–oxide interfaces leveraged the dual benefits of metallic Fe for catalytic activity and the perovskite matrix for structural stability. With ultra-high current efficiency (98%) and prolonged operational durability, as shown in Figure 9f (70 h, 7 redox cycles), their study established a compelling strategy for CO_2_ and CH_4_ reforming.

Xu et al. [104] extended the application of exsolved Fe nanoparticles to steam electrolysis, emphasizing their role in enhancing H₂ production. The study synthesized La_0.6_Sr_0.4_Fe_x_O_3₋δ_ (LSF_x_, x = 0.8–1.2) perovskites, systematically varying the Fe content to optimize the exsolution process. The LSF_1.1_ cathode demonstrated exceptional performance with a polarization resistance of 0.31 Ω·cm^2^ and nearly 100% current efficiency at 850 °C. Their work underscores the importance of stoichiometric tuning to achieve stable and active metal–oxide interfaces, contributing to robust long-term operation.

**Table 7:** Properties of iron exsolution materials.

Exsolved Metal	Electrode Composition	T [°C]	Gas Composition	Potential [V]	Current Density [A cm^−2^]	Ref.
Fe	Fe-RPLSF	850	Pure CO_2_	1.5	1.92	[95]
Fe	(Pr_0.5_Ba_0.5_)_1.8_Fe_1.8_Mn_0.2_O_5-δ_	850	Pure CO_2_	1.8	1.6	[100]
Fe	Sr_2_Fe_1.4_Zn_0.1_Mo_0.5_O_6-δ_	850	High purity CO_2_	1.6	2.74	[96]
Fe	Sr_2_Fe_1.5+x_Mo_0.5_O_6-δ_	850	CO_2_	1.6	~0.70	[101]
Fe	La_0.6_Sr_0.4_Mn_0.2_Fe_0.8_O_3-δ_	850	30% CO/CO_2_	1.5	2.04	[102]
Fe	Sm_0.2_Ce_0.8_O_2-δ_	800	50% CO/CO_2_	1.5	1.35	[103]
Fe	(La_0.75_Sr_0.25_)_0.9_(Cr_0.5_Mn_0.5_)_0.9_Fe_0.1_O_3-δ_	850	Anode: CH_4_ Cathode: CO_2_	1.2	0.40	[97]
Fe	La_0.6_Sr_0.4_Fe_x_O_3-δ_	850	Steam	1.6	0.74	[104]

In conclusion, the exsolution process of single-metal nanoparticles offers high controllability and significantly increases the oxygen vacancy concentration in the oxide substrate [105]. The nanoparticles formed after exsolution can reduce the activation energy of the electrode reaction, thereby improving the energy conversion efficiency of solid oxide cells (SOCs) and extending the operational lifespan of electrodes.

### 3.2. Exsolution of Alloy Nanoparticles

In solid oxide cells (SOCs), the exsolution of alloy nanoparticles offers several advantages compared to the exsolution of single-metal nanoparticles. The exsolution process facilitates the adjustment of nanoparticle size, morphology, and distribution, resulting in a more uniform catalyst dispersion on the electrode surface. Furthermore, the synergistic interactions between alloy components can enhance the kinetics of redox reactions and accelerate the formation of oxygen vacancies. By fine-tuning the composition and ratio of the constituent metals, the catalytic performance can be precisely optimized. Additionally, the mutual stabilization of alloy constituents mitigates sintering, thereby enhancing structural stability and maintaining electrode activity over extended operational periods [106,107]. This section of this review summarizes recent advancements in the application of Co-Fe, Ni-Fe, and other common alloy systems in SOCs.

#### 3.2.1. Co-Fe Alloy Nanoparticles

Co-Fe alloys, characterized by their superior catalytic activity, structural stability, and resistance to carbon deposition, exhibit significant potential for application in solid oxide cells (SOCs) [108]. This section provides an overview of the current status and prospects of Co-Fe alloys in applications such as CO₂ electrolysis and methane conversion.

**Figure 10:**
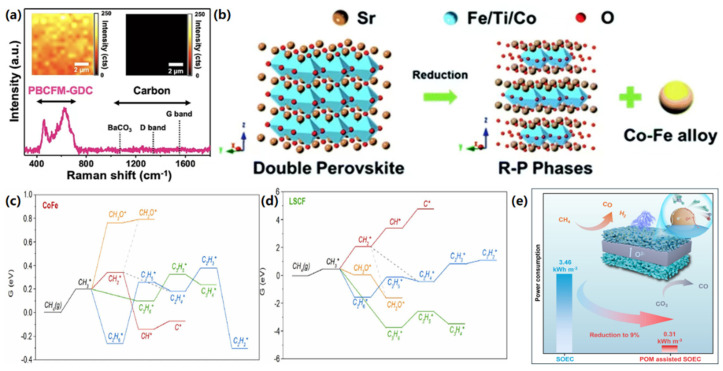
(**a**) Raman spectroscopy data collected from the PBCFM−GDC fuel electrode after a stability test under CO_2_ electrolysis conditions; the inset shows the corresponding Raman mapping of PBCFM−GDC and carbon on the electrode surface [109]. Reproduced with permission from [109]. (**b**) Illustration of the structural transformation of STCF during the exsolution process [112]. Reproduced with permission from [112]. Density functional theory (DFT)-evaluated free energy diagrams for surface reactions on (**c**) Co−Fe and (**d**) LSCF (110) surfaces at 0 V RHE [116]. Reproduced with permission from [116]. (**e**) In situ exsolved CoFe alloy nanoparticles on perovskite anodes exhibit excellent and stable performance in CH₄ reforming [117]. Reproduced with permission from [117].

The exsolution of Co-Fe alloys has emerged as a critical strategy for enhancing the performance of carbon dioxide (CO_2_) electrolysis, particularly on perovskite oxide-based electrodes. For instance, Kyung et al. [109] developed a Pr_4/3_Ba_2/3_Co_2/3_Fe_2/3_Mn_2/3_O_5+δ_ (PBCFM) perovskite material, where doping with Fe and Mn significantly improved the structural stability of PrBaCo_2_O_5+δ_ and prevented its phase decomposition in a pure CO_2_ atmosphere. Co-Fe alloy nanoparticles precipitated on the surface of PBCFM under H_2_ reduction conditions significantly enhanced CO_2_ electroreduction, achieving a high current density of 3.76 A·cm^−2^ at 1.5 V. Additionally, as illustrated in Figure 10a, no carbon deposition was observed during long-term operation tests at 1.2 V. Similarly, Zhang et al. [110] prepared a CoFe-based perovskite electrode Pr_0.4_Sr_0.6_Co_0.125_Fe_0.75_Mo_0.125_O_3-δ_ (PSFCM). Under reducing conditions, CoFe alloy nanoparticles precipitated on the electrode exhibited improved stability and reversibility through multiple redox cycles. The biphasic CoFe-PSFCM-Ce_0.8_Sm_0.2_O_1.9_ (SDC) electrode achieved an impressive current density of 1.42 A·cm^−2^ at 1.6 V, significantly surpassing the original PSFCM electrode’s performance of 0.83 A·cm^−2^. DRT resolution of the EIS during 270 h of operation showed that no significant deterioration or loss of performance occurred during the endurance test. Furthermore, Sr_2_FeMo_1-x_Co_x_O_6-δ_ electrocatalysts with high Co doping (x = 0.45), developed by Xi et al. [111], demonstrated excellent coking resistance and long-term stability. This superior performance may be attributed to the unique simultaneous in situ formation of heterogeneous nano-alloyed oxide structures and electron–ion hybrid conducting perovskites with defect-rich matrices.

Sun et al. [112] also investigated the reduction of the stoichiometric double perovskite oxide Sr_2_Ti_0.8_Co_0.2_FeO_6_, leading to the formation of Co-Fe alloy nanoparticles on the electrode surface, as illustrated in Figure 10b. This electrode demonstrated superior performance and stability in both solid oxide electrolysis cell (SOEC) and solid oxide fuel cell (SOFC) dual-mode operations at 800 °C in a pure CO_2_ environment. After 100 h, the Co-Fe alloy nanoparticles retained their initial structure without agglomeration, and no carbon deposition was generated at the electrode. The researchers suggested that the slight decay of SOEC performance may be related to the alteration of the microstructure of the electrode/electrolyte interface in the pure CO_2_ high-temperature test environment. Co-doped oxides and metal nanoparticles on the surface of perovskite oxides (cathode) exhibit enhanced cell electrolytic performance compared to singly doped counterparts [113,114]. Moreover, the Ce/Co co-doped SrFeO3-δ (SF) electrode developed by Ni et al. [115] exhibited improved CO_2_ adsorption properties relative to single-doped variants, indicating its potential as a symmetric electrode for CO_2_ electrolysis. These studies highlight the critical role of Co-Fe alloy exsolution in optimizing electrocatalytic performance for CO_2_ reduction, providing a foundation for the development of efficient and stable electrodes in CO₂ electrolysis applications.

The exsolution of Co-Fe alloys plays a pivotal role in enhancing the performance of anodes for reactions such as the oxidative coupling of methane (OCM) and partial oxidation of methane (POM) in solid oxide electrolysis cells (SOECs). Qin et al. [118] developed Sr_1.95_Fe_1.4_Co_0.1_Mo_0.5_O_6-δ_ perovskite oxides, which exhibited a heterogeneous structure with in situ exsolution CoFe alloys and abundant oxygen vacancies under reduction conditions. The CoFe@SFCoM anode demonstrated excellent performance, achieving a current density of 1.89 A·cm^−2^, an ethane conversion rate of 36.4%, and ethylene selectivity of 94.5% at 1.6 V and 800 °C. After a 50 h stability test, no significant changes in electrolytic current density or ethylene yield were observed, highlighting its potential for high-efficiency ethane electrocatalytic production. Similarly, Jaesung Kim et al. [116] achieved enhanced electrocatalytic activity for OCM by exsolving CoFe nanoparticles from La-Sr-Co-Fe perovskite (LSCF), thereby improving surface reactivity. The reduced LSCF exhibited a C_2_^+^ hydrocarbon selectivity of 63% and a C_3_H_6_ selectivity of 10.2%. Density functional theory (DFT) calculations emphasized the critical role of Co-Fe nanoparticles in facilitating selective OCM pathways, as illustrated in Figure 10c,d. Furthermore, the study demonstrated that controlling exsolution and reoxidation cycles mitigates nanoparticle sintering while optimizing oxygen supply and suppressing deep CH_4_ oxidation, thereby further improving selectivity.

In a subsequent study, Guo et al. [117] investigated the in situ exsolution of CoFe alloy nanoparticles on La-Sr-Ti-Fe-Co perovskite anodes for the partial oxidation of methane (POM) in solid oxide electrolysis cells (SOECs). By optimizing the B-site exsolution energy through precise Co/Fe doping, the LSTFC_2_ anode achieved a high nanoparticle density of 1087 particles/mm^2^ and a CH_4_ conversion rate of 86.9%, with CO selectivity reaching 90.1%. The anode demonstrated stable operation over 1250 h, maintaining CO selectivity above 95% and reducing the energy consumption for CO production from 3.46 to 0.31 kWh/m^3^, as illustrated in Figure 10e. These results underscore the potential of CoFe alloy exsolution in enhancing both the performance and durability of SOECs for hydrocarbon conversions, although the impact of byproduct formation, such as coke deposition, on long-term stability was not fully addressed. Collectively, these studies highlight the critical role of Co-Fe alloy exsolution in optimizing the electrocatalytic performance of anodes for hydrocarbon conversions.

**Table 8:** Properties of Co-Fe exsolution materials.

Exsolved Metal	Electrode Composition	T [°C]	Gas Composition	Potential [V]	Current Density [A cm^−2^]	Ref.
Co-Fe	Pr_4/3_Ba_2/3_Co_2/3_Fe_2/3_Mn_2/3_O_5+δ_	850	Pure CO_2_	1.5	3.76	[109]
Co-Fe	Pr_0.4_Sr_0.6_Co_0.125_Fe_0.75_Mo_0.125_O_3-δ_	800	Anode: air Cathode: H_2_/CO_2_	1.6	1.42	[110]
Co-Fe	Sr_2_FeMo_1-x_Co_x_O_6−δ_ (x = 0, 0.15, 0.25, 0.45)	800	Pure CO_2_	1.5	2.00	[111]
Co-Fe	Sr_2_Ti_0.8_Co_0.2_FeO_6-δ_	800	Pure CO_2_	1.6	1.26	[112]
Co-Fe	Sr_1−x_Ce_x_Fe_1−y_Co_y_O_3_	800	Pure CO_2_	1.3	1.30	[115]
Co-Fe	Sr_1.95_Fe_1.4_Co_0.1_Mo_0.5_O_6-δ_	800	Anode: C_2_H_6_ Cathode: CO_2_	1.6	1.89	[118]
Co-Fe	La_0.7_Sr_0.2_Co_0.2_Fe_0.8_O_3_ (LSCF)	850	Pure CH_4_	0.25	0.15	[116]
Co-Fe	La_0.6_Sr_0.4_Ti_0.3_Fe_0.5_Co_0.2_O_3-δ_	800	Anode: 40% CH_4_ + 55% Ar + 5%N_2_ Cathode: 95% CO_2_-5% Ar	1.5	~0.275	[117]

#### 3.2.2. Ni-Fe Alloy Nanoparticles

Ni-Fe alloys exhibit exceptional electrocatalytic performance, particularly under H₂ or CH_4_ fuel conditions, characterized by high catalytic activity and low electrode reaction impedance. Compared to pure nickel or other Ni-based alloys, Ni-Fe alloys demonstrate superior oxidation resistance and corrosion tolerance in high-temperature reducing environments. The incorporation of Fe plays a pivotal role in modifying the surface chemical properties of Ni, effectively mitigating carbon deposition issues [119]. Additionally, the thermal expansion coefficient of Ni-Fe alloys is more closely aligned with that of solid oxide cell materials, thereby enhancing electrode stability and prolonging operational lifespan [120]. Collectively, these attributes highlight the potential of Ni-Fe alloys as highly efficient and durable materials for application in solid oxide cells.

**Figure 11:**
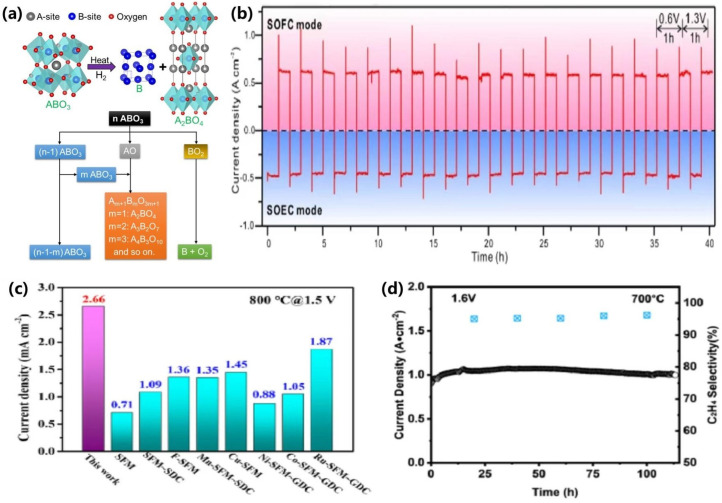
(**a**) Schematic illustration of the concurrent formation of B−site metal phase and Ruddlesden−Popper phase during the exsolution process [121]. Reproduced with permission from [121]. (**b**) Short−term durability test results for the NiFe−SFM cell at 800 °C, involving 20 reversible operation cycles with 0.6 V under SOFC mode and 1.3 V under SOEC mode [122]. Reproduced with permission from [122]. (**c**) Comparison of current density at 800 °C and 1.5 V using various SFM-based cathodes [123]. Reproduced with permission from [123]. (**d**) Long−term stability evaluation of full cells equipped with the NCMF anode [124]. Reproduced with permission from [124].

The exsolution of Ni-Fe nanoparticles during CO_2_ electrolysis significantly enhances the catalytic properties of solid oxide cell (SOC) materials. This phenomenon is closely associated with the formation of oxygen vacancies, which promote surface reactions and thereby boost the catalytic activity of parent perovskite compounds. For instance, Dhruba J. Deka et al. [121] demonstrated that reducing Ni-doped (La,Sr)FeO_3_ perovskite at 400 °C—the lowest temperature at which nanoparticle exsolution was observed—resulted in a surface enriched with B-site metal nanoparticles and oxygen vacancies (as schematically illustrated in Figure 11a). Importantly, this exsolution not only enhanced the CO_2_ reduction activity of the LSNF cathode but also increased the electrical energy consumption required for the process. This finding underscores the potential for future electrolyte advancements that could enable SOCs to operate at lower temperatures while achieving improved performance. Similarly, Li et al. [122] developed a bifunctional fuel electrode composed of an SFM scaffold with epitaxially grown NiFe nanoparticles. This innovative design exhibited accelerated oxygen exchange kinetics and facilitated CO/CO_2_ activation. In their study, DRT analysis was employed to effectively separate multiple overlapping time constants in EIS occurring at the electrode. Moreover, as shown in Figure 11b, the electrode demonstrated remarkable stability during reversible mode switching over 20 cycles, offering a promising solution for robust reversible solid oxide cell (RSOC) applications.

The electronegativity of fluorine (F, λ = 3.98) is significantly higher than that of oxygen (O, λ = 3.44). Partial substitution of oxygen with doped fluorine weakens the metal–oxygen bond, thereby activating lattice oxygen and promoting the formation of oxygen vacancies [125]. These oxygen vacancies play a critical role in facilitating the diffusion of B-site cations and enhancing exsolution reactions. Additionally, surface oxygen vacancies effectively anchor exsolved nanoparticles, preventing their agglomeration and improving stability [126]. Xia et al. [123] first proposed a co-doping strategy involving Ni and F to enhance the exsolution reaction, leading to the development of a novel cathode material: Ni-Fe alloy nanoparticles encapsulated within fluorine-doped Sr_2_Fe_1.5_Mo_0.5_O_6-δ_ (SFM). The combined effects of F-doping and Ni-Fe exsolution resulted in a 2.4-fold increase in CO_2_ adsorption. Moreover, an exceptionally high current density of 2.66 A·cm^−2^ was achieved at 800 °C and 1.5 V, as illustrated in Figure 11c, with the material maintaining stability for over 140 h. SEM cross-sectional morphology after long-term testing observed that both the porous cathode and anode were tightly adhered to the dense LSGM electrolyte layer, indicating good structural stability of the cell; no carbon deposition was detected by Raman spectroscopy. Furthermore, La_0.6_Sr_0.4_Ni_0.2_Fe_0.75_Mo_0.05_O_3-δ_ (LSNFM) electrodes facilitated the reduction of FeNi_3_ nanoparticles, leading to increased electrolytic current density and reduced interfacial polarization resistance [127]. Xia and his team have made significant contributions to this field, advancing the development of efficient cathode materials for CO_2_ electrolysis.

Further studies have also underscored the significance of metal alloying and doping in enhancing CO_2_ electrolysis performance. Ansari et al. [128] reported that LCFCrN perovskites exhibited promising properties, with Fe-Ni alloy nanoparticles improving both CO oxidation and CO_2_ reduction kinetics, making these materials suitable for reversible solid oxide cells (RSOCs). Xu et al. [129] compared NiFe and NiCo co-doped perovskite fibers, finding that NiFe co-doped fibers demonstrated faster oxygen vacancy formation and stronger CO_2_ interactions with Fe-containing clusters. Li et al. [130] synthesized Ni-doped Pr_0.4_Sr_0.6_Fe_0.9_Mo_0.1_O_3_ perovskites under reducing conditions, resulting in the formation of NiFe_10.8_ nanoparticles. The reduced Pr_0.4_Sr_0.6_Fe_0.9_Mo_0.1_O_3_ exhibited the highest concentration of oxygen vacancies, superior CO_2_ adsorption capabilities, and exceptional catalytic activity, positioning it as a promising candidate for CO_2_ electrolysis applications. Collectively, these studies highlight the critical role of Ni-Fe nanoparticle exsolution in improving CO_2_ electrolysis efficiency and stability through the utilization of oxygen vacancies, surface modifications, and alloying strategies.

Tian et al. [131] introduced Pr_0.5_Ba_0.5_Fe_0.8_Ni_0.2_O_3-δ_ (PBFN) as a high-performance electrode material for symmetric solid oxide cells (SSOCs), leveraging the exsolution of Ni-Fe alloy nanoparticles and the phase transition from single cubic perovskite to A-site ordered double perovskite. This dual mechanism significantly enhanced the oxygen vacancy concentration and the number of catalytically active sites, thereby improving electrochemical activity for various reactions, including oxygen reduction, oxygen evolution, CO_2_ reduction, and hydrogen oxidation. The PBFN-based SSOCs exhibited robust performance and stability in both fuel cell and electrolysis modes, demonstrating high power density, current density, and excellent durability. Their work underscores the transformative potential of phase transitions and nanoparticle exsolution in advancing SSOC electrode materials. In a related study, Zhang et al. [124] presented a multiphase nanocomposite anode, Ce_0.6_Mn_0.3_Fe_0.1_O_2-δ_/NiFe/MnO_x_ (CMF-NiFe-MnO_x_), specifically designed for the electrooxidative dehydrogenation of ethane (EODHE) in solid oxide electrolysis cells (SOECs). The dual active-site design, combining NiFe alloy nanoparticles for dehydrogenation and MnO_x_ nanoparticles for selective oxidation, along with optimized oxygen vacancies in the matrix, enabled efficient ethane conversion with high ethylene selectivity. Operating at intermediate temperatures, this material achieved significant structural and catalytic stability over prolonged operation (120 h), as shown in Figure 11d. This innovative approach advances the design of SOEC anodes, offering not only high catalytic activity but also operational durability, positioning it as a promising candidate for clean and efficient alkane conversion technologies. Although the ethylene selectivity is high, the study does not extensively address potential byproducts or long-term catalyst deactivation mechanisms under varying operating conditions, which are crucial for industrial applications.

**Table 9:** Properties of Ni-Fe exsolution materials.

Exsolved Metal	Electrode Composition	T [°C]	Gas Composition	Potential [V]	Current Density [A cm^−2^]	Ref.
Ni-Fe	(La,Sr)FeO_3_	800	40% CO_2_/He	1.1	0.29	[121]
Ni-Fe	Sr_2_Fe_1.5_Mo_0.5_O_6−δ_	800	CO−CO_2_ (7:3)	1.5	1.15	[122]
Ni-Fe	Sr_2_Fe_1.5_Mo_0.5_O_6-δ_	800	Pure CO_2_	1.5	2.66	[123]
Ni-Fe	La_0.6_Sr_0.4_Ni_0.2_Fe_0.75_Mo_0.05_O_3-δ_	800	Pure CO_2_	1.5	0.59	[127]
Ni-Fe	La_0.3_Ca_0.7_Fe_0.7_Cr_0.3_O_3-δ_	800	Pure CO_2_	1.6	0.65	[128]
Ni-Fe	La_0.52_Ca_0.28_Ni_0.04_Fe_0.04_Ti_0.92_O_3_/La_0.52_Ca_0.28_Ni_0.03_Co_0.03_Ti_0.94_O_3_	900	Pure CO_2_	1.6	0.75	[129]
Ni-Fe	Pr_0.4_Sr_0.6_Fe_0.9_M_o0.1_O_3_	800	Pure CO_2_	1.5	1.05	[130]
Ni-Fe	Pr_0.5_Ba_0.5_Fe_0.8_Ni_0.2_O_3-δ_	800	Pure CO_2_	1.4	0.28	[131]
Ni-Fe	Ce_0.6_Mn_0.3_Fe_0.1_O_2−δ_-NiFe-MnO_x_	700	Fuel electrode: CO_2_ + H_2_ Hydrogen electrode: 50% C_2_H_6_/Ar	1.8	1.62	[124]

#### 3.2.3. Others Alloy Nanoparticles

**Figure 12:**
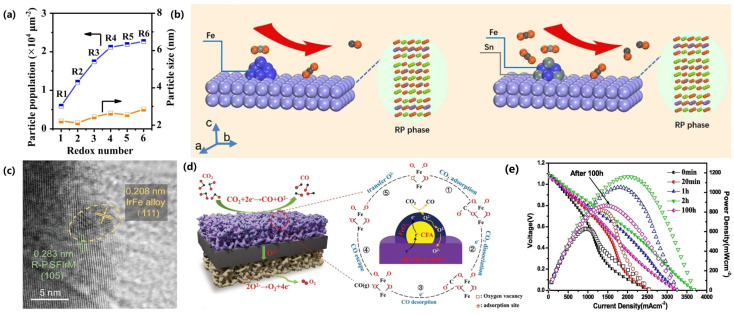
(**a**) Population and size distribution of metal nanoparticles (NPs) exsolved after various redox manipulations [133]. Reproduced with permission from [133]. (**b**) Schematic illustration of CO_2_ adsorption on Fe nanoparticles and Fe−Sn alloy nanoparticles [136]. Reproduced with permission from [136]. (**c**) Characteristics of the exsolved IrFe@SFIrM interfaces as observed in bright−field STEM images [137]. Reproduced with permission from [137]. (**d**) Schematic illustration of the CO_2_ electrolysis process on a high−entropy perovskite cathode coated with core−shell CuFe alloy@FeOx (HE−PSCFMMN-CFA@FeO) nanoparticles, highlighting the reaction process of CO_2_ on the oxide shell (FeO) of the nanoparticles. The orange box represents oxygen vacancies, and * denotes adsorption sites [139]. Reproduced with permission from [139]. (**e**) Current–voltage (I−V) and current–power (I−P) curves for the cell with the SFMC0.1 fuel electrode at 800 °C, under different reduction times [140]. Reproduced with permission from [140].

In addition to the widely used Co-Fe and Ni-Fe alloys, various other metal alloys also play crucial roles in solid oxide cell (SOC) technology applications. Ru-Fe alloys in solid oxide electrolysis cells (SOECs) for CO_2_ reduction have been shown to enhance both catalytic performance and cathode stability. Zhang et al. [132] demonstrated that Ru-doped perovskite Pr_0.4_Sr_0.6_Fe_0.9_Mo_0.1_O_3-δ_ significantly improved the current density and Faradaic efficiency of CO_2_ reduction, enhancing overall performance by 109%. They identified that in situ exsolved Fe-Ru alloy nanocatalysts facilitate CO_2_ dissociation, making them more active for electrochemical CO_2_ reduction. Similarly, Lv et al. [133] promoted the exsolution of RuFe alloy nanoparticles on Sr_2_Fe_1.4_Ru_0.1_Mo_0.5_O_6−δ_ through repeated redox manipulations, resulting in a 74.6% increase in current density during CO_2_ electrolysis. Notably, the redox process did not alter the size of the exsolved metal nanoparticles, as illustrated in Figure 12a. This provides new ideas for constructing rich metal–oxide catalytic interfaces on the surface of perovskite materials through repetitive redox modulation strategies. However, these systems often face complex exsolution dynamics and require precise control of the redox processes to optimize the catalyst interface, which remains a practical challenge.

Ni-Co alloy nanoparticles have also shown promise in enhancing CO_2_ reduction. Liu et al. [134] exsolved Ni-Co alloys from layered perovskite oxide (LSTNC-r), achieving a high current density of 1.53 A·cm^−2^ at 850 °C, along with significant improvements in CO_2_ reduction efficiency. Ma et al. [135] further emphasized the critical role of metal–oxide interfaces, demonstrating that Ni-Cu alloy nanoparticles on perovskite electrodes achieved a Faradaic efficiency of 97.7% for CO production. Fe-Sn alloys offer another promising approach. Lv et al. [136] reported that Sn-doped perovskite oxides Sr_1.95_Fe_1.4_Sn_0.1_Mo_0.5_O_6−δ_, with in situ exsolution of Fe-Sn alloy nanoparticles for CO_2_ electrolysis, exhibited improved CO_2_ adsorption performance, as illustrated in Figure 12b. The alloy exsolution enhanced current density in CO_2_ electrolysis, achieving 3.269 A·cm^−2^, and demonstrated good stability over 200 h of operation. The enhanced CO_2_ chemisorption and catalytic activity of this system underscore its potential, but further understanding of nanoparticle behavior under extended operational conditions is needed for practical application.

The concept of in situ electrochemical reconstruction of cathodes during CO_2_ electrolysis, as demonstrated by Shen et al. [137] and Xi et al. [138], holds significant potential for enhancing the performance of solid oxide electrolysis cells (SOECs). These studies focused on the dynamic reconstruction of metal–oxide interfaces under operational conditions. Shen et al. identified that iridium-doped Sr_2_Fe_1.45_Ir_0.05_Mo_0.5_O_6-δ_ (SFIrM) perovskite exhibited a dynamic electrochemical reconstruction feature during CO_2_ electrolysis, characterized by the abundant exsolution of highly dispersed IrFe alloy nanoparticles on the SFIrM surface, as illustrated in Figure 12c, leading to substantial improvements in current density and Faradaic efficiency. Xi et al. demonstrated that the dynamic reconstruction of Fe-Cu alloy nanoparticles during CO_2_ electrolysis resulted in high electrochemical performance, achieving a current density of 1.7 A cm^−2^ with a Faradaic efficiency of 95.2%. Wang et al. [139] introduced a high-entropy perovskite cathode coated with core–shell CuFe alloy@FeOx (CFA@FeO) nanoparticles, which exhibited remarkable CO_2_ reduction activity (1.95 A cm^−2^) and long-term stability (200 h). Based on DFT calculations, it is speculated that the mechanism of electrocatalytic CO_2_ reduction for the CFA@FeO HE-PSCFMMN cathode, as illustrated in Figure 12d, involves the promotion of additional oxygen vacancies, thereby enhancing the kinetics of CO_2_ adsorption and reduction. While these findings are promising, the challenge remains in precisely controlling the dynamic changes at the metal–oxide interface, as uncontrolled reconstruction processes can lead to instability.

Innovative design strategies, such as the incorporation of core–shell structures and high-entropy perovskites, have greatly advanced the efficiency of CO_2_ reduction processes. López-García et al. [141] demonstrated the tunability of ternary alloy nanoparticles in solid oxide electrolysis cells (SOECs), emphasizing the potential to optimize the composition of metal alloys to enhance catalytic activity and improve nanoparticle stability. However, these advanced configurations often present challenges related to synthesis complexity, high costs, and scalability limitations. In a complementary vein, synergistic control of operational conditions, such as applied potential and temperature, has been shown to optimize ethane conversion and ethylene selectivity [142]. Zhang et al. [143] further explored the potential of CeO_2_-based metal oxide materials for electrochemical oxidative dehydrogenation of ethane (C_2_H_6_) within solid oxide electrolyzer (SOE) devices. By doping Ni/Cu alloys into CeO_2_ and utilizing a reducing H_2_/Ar atmosphere, the formation of metal–oxide interfaces markedly enhanced catalyst performance. The material achieved a current density of 0.51 A cm^−2^, a C_2_H_6_ conversion rate of 38.9%, and an ethylene yield of 24.9%, maintaining impressive stability over 100 h. These findings highlight the role of metal–oxide interfaces in promoting ethane activation, supported by density functional theory (DFT) calculations. While their work innovatively leverages in situ out-of-solution metal oxide interfaces, the economic feasibility of scaling this technology for industrial application remains an open question. Additionally, Wu et al. [140] examined the exsolution process of copper–iron (Cu-Fe) nanoparticles in Sr_1.9_Fe_1.5_Mo_0.5_O_6-δ_ (SFMC) materials, targeting the improvement in performance and durability of ceramic anodes in solid oxide fuel cells (SOFC). Their study demonstrated that Cu nanoparticles form a protective core–shell structure around Fe nanoparticles via adjusting reduction conditions, particularly with the inclusion of water vapor, thus preventing agglomeration at high temperatures. This modification resulted in a notable increase in power density from 0.63 to 1.2 W cm^−2^, as illustrated in Figure 12e, attributed to the controlled morphology of exsolved nanoparticles and phase transitions during reduction. The approach of using water vapor to control nanoparticle morphology at elevated temperatures presents a novel strategy for enhancing anode performance. However, the long-term stability of the Cu-Fe composite in real-world SOFC applications has not been fully addressed. Further investigation into the durability of the core–shell nanoparticles and their performance under extended operational conditions is essential to assess the practical applicability of this approach in industrial settings.

**Table 10:** Properties of other alloy exsolution materials.

Exsolved Metal	Electrode Composition	T [°C]	Gas Composition	Potential [V]	Current Density [A cm^−2^]	Ref.
Ru-Fe	Pr_0.4_Sr_0.6_Fe_0.9_Mo_0.1_O_3-δ_	800	Pure CO_2_	2.0	1.48	[132]
Ru-Fe	Sr_2_Fe_1.4_Ru_0.1_Mo_0.5_O_6−δ_	800	95% CO_2_ + 5% N_2_	1.6	~1.35	[133]
Ni-Co	(La_4_Sr_4_)_0.9_Ti_7.2_Ni_0.4_Co_0.4_O_2−δ_	850	Pure CO_2_	2.0	1.53	[134]
Ni-Cu	La_0.7_Sr_0.3_Cr_0.5_Mn_0.5_(NiCu)_x_O_3−δ_	850	Pure CO_2_	1.6	0.68	[135]
Fe-Sn	Sr_1.95_Fe_1.4_Sn_0.1_Mo_0.5_O_6-δ_	800	Pure CO_2_	1.8	3.269	[136]
Ir-Fe	Sr_2_Fe_1.45_Ir_0.05_Mo_0.5_O_6-δ_	800	95% CO_2_/5% N_2_	1.6	1.46	[137]
Fe-Cu	Sr_2_Fe_1.25_Cu_0.25_Mo_0.5_O_6-δ_	800	Pure CO_2_	1.4	1.70	[138]
Fe-Cu	Pr_0.8_Sr_1.2_(CuFe)_0.4_Mo_0.2_Mn_0.2_Nb_0.2_O_4-δ_	800	Pure CO_2_	1.5	1.95	[139]
Cu-Fe	Sr_1.9_Fe_1.5_Mo_0.4_Cu_0.1_O_6-δ_	800	Cathode: wet H_2_ (3% H_2_O) Anode: air	0.99	1.2W cm^−2^	[140]
Fe−Co−Ni	Sr_x_FeCo_0.2_Ni_0.2_Mn_0.1_Mo_0.5_O_6−δ_	800	Cathode: 15%CO_2_/Ar Anode: air (60 mLmin^−1^)	1.2	0.19	[141]
Ni-Cu	Ce_0.9_(Ni_x_Cu_1-x_)_0.1_O_2-δ_ (x = 0–1)	700	C_2_H_4_	1.0	0.51	[143]

In summary, the exsolution of alloy nanoparticles, including Ru-Fe, Co-Fe, Fe-Sn, and others, holds significant promise for enhancing the performance of solid oxide electrolysis cells (SOECs) in CO_2_ reduction. Key benefits include improved current densities, Faradaic efficiencies, and stability during extended operations.

## 4. Topotactic Ion Exchange

The application of topotactic ion exchange in exsolution offers a novel approach to facilitate extensive cation exsolution [144]. Under highly reducing conditions, B-site metals (e.g., Co, Fe) migrate significantly from the substrate to the surface, forming stable metal nanoparticles. This exsolution process enables the controlled synthesis of metal nanoparticles without introducing cation vacancies into the host lattice, thereby preserving the structural integrity of the parent metal oxide and markedly enhancing the catalytic activity of perovskites [145]. Topological ion exchange usually involves both infiltration and exsolution processes, which we discuss briefly here Table 11 summarizes the topological ion exchange strategies for the SOCs in this section

Sangwook Joo et al. [146] were the first to demonstrate the practical application of the topotactic ion exchange/exsolution strategy. In their work, infiltrated Fe guest cations were exchanged with Co host cations in PrBaMn_1.7_Co_0.3_O_5+δ_ (PBMCo), leading to the formation of CoFe alloy nanoparticles. An electrolyte-supported cell utilizing the PBMCo-12-Fe anode (with 12 wt% Fe infiltration) achieved a maximum power density of 1.834 W cm^−2^ at 800 °C under a humidified hydrogen atmosphere. The study further revealed that the exsolution-promoting effect tended to saturate beyond a specific infiltration threshold. This strategy underscores the unique potential of topotactic ion exchange for selectively exsolving catalytic nanoparticles from oxide materials. Also, they [147] introduced Fe guest ions into a PrBaMn_1.7_Ni_0.3_O_5+δ_ (PBMNI) perovskite host for methane dry reforming, which resulted in a higher number of Ni-Fe alloy nanoparticles by topotactic exsolution. The dispersed Ni-Fe alloy metal particles improved the catalytic activity with stable performance compared to pristine PBMNI without alloy catalysts. These findings suggest that topology is an effective means of catalyzing synthesis in gas-phase reactions by generating bimetallic nanoparticles.

A novel three-phase composite cathode, synthesized by in situ ion topology engineering, improved the performance of Proton ceramic fuel cells (PCFC), as demonstrated by Tang et al. [148]. By introducing transition metal V into BaCe_0.25_Fe_0.7_5O_3-***δ***_ (BCF), BaFe_2_O_4_ nanoparticles were formed on the cathode surface via Fe-Ba-V ion exchange, and BaCeO_3_-BaFe_1-x_V_x_O_3_ cocatalytic boundaries were also formed. The efficient surface diffusion and oxygen ion transport properties of the material were verified by DRT analysis. An impressive power density of 1.73 W cm^−2^ at 650 °C was achieved using BCF-V as the cathode, and stable operation was maintained for more than 200 h at 600 °C. This makes it a good candidate for proton conductivity.

By introducing Fe guest cations, LV et al. [149] caused a host material to form a perovskite structure with a stoichiometric ratio of Pr_0.7_Sr_0.3_Cr_0.9_(FeNi)_0.1_O_3-δ_ and triggered the exsolution of Ni cations from the crystal lattice while maintaining structural stability. The scholars propose that the number of FeNi alloy nanoparticles formed gradually increased with an increase in Fe loading. However, it is worth noting that the excessive introduction of FeO_x_ guest components exacerbated the local segregation phenomenon, which may lead to pore clogging and accelerated property degradation. Experimental studies together with DFT calculations revealed that the enhanced catalytic activity of the electrode performance stemmed from the unique CO_2_ adsorption and activation capacity of the metal/calcite interface. In addition, Guntae Kim’s team [150] developed active and durable catalysts through topological alternatives combined with atomic layer deposition (ALD).

**Table 11:** Properties of topotactic ion exchange.

Exsolved Metal	Electrode Composition	T [°C]	Gas Composition	Potential [V]	Current Density [A cm^−2^]	Ref.
CO-Fe	PrBaMn_1.7_Co_0.3_O_5+δ_ + 12 wt% Fe -GDC	800	97% H_2_/3% H_2_O	~0.90	1.20	[146]
Fe-Ni	PrBaMn_1.7_Ni_0.3_O_5+δ_ + 12 wt% Fe -GDC	800	97% H_2_/3% H_2_O	0.80	~1.30	[147]
BaFe_2_O_4_	BaCe_0.25_Fe_0.75_O_3-*δ*_-xV_2_O_3_	600	50% N_2_/50% O_2_	0.95	0.20	[148]
Fe-Ni	La_0.6_Sr_0.4_Co_0.2_Fe_0.8_O_3-δ_-GDC	800	CO_2_	1.49	1.60	[149]
Fe-Ni	La_0.6_Sr_0.2_Ti_0.85_Ni_0.15_O_3-δ_ + Fe_2_O_3_	700	10% CO_2_-10% CH_4_-80% He	/	/	[150]

In conclusion, the selective exsolution process by topological ion exchange is an interesting study. This approach, which takes into account how the internal spatial structure, porosity, and surface properties of a material affect the adsorption, exchange, and release of ions [151], has a wide range of applications in SOCs.

## 5. Summary and Perspectives

Solid oxide cells (SOCs) are fully solid-state devices for energy conversion and storage, distinguished by their high-temperature ionic conduction capabilities. This characteristic endows SOCs with several advantages, including high efficiency, environmental sustainability, and economic feasibility, making them a prime candidate for next-generation energy technologies. Despite considerable advancements in SOCs over the past decade, challenges remain, particularly regarding the electrochemical performance of electrodes at reduced temperatures. Nanoparticles (NPs), characterized by their high specific surface areas and numerous active sites, offer promising solutions through their nanoscale effects. In SOCs incorporating NPs, various electrochemical reactions—such as CO_2_ electrolysis, H_2_O electrolysis, CO_2_/H_2_O co-electrolysis, and methane/ethane electrochemical oxidation dehydrogenation—are extensively researched. The development of nanotechnologies that enable the efficient synthesis of NPs and their integration into SOCs is critical for both fundamental research and commercial applications. This review examines recent progress in nanoengineering electrodes using infiltration and exsolution, two widely adopted methods for fabricating nanoparticles. The infiltration technique has proven effective for in situ NP formation but faces challenges related to the weak bonding between the deposited NPs and the substrate. Conversely, the cutting-edge approach of in situ exsolution offers a robust solution for generating highly active NPs. The strong interaction between the material matrix and NPs enhances electrochemical activities by influencing internal ionic diffusion and electronic conduction, thereby improving electrode performance in terms of gas molecule adsorption and oxygen reduction. Overall, both methods provide extensive opportunities to expand the range of catalyst types, creating new possibilities for enhancing the functionality and efficiency of SOCs.

Significant advancements in common nanotechnology over the past decade have not fully resolved many critical challenges in solid oxide cells (SOCs). Therefore, it is imperative to address specific research objectives related to nanoparticle (NP) infiltration and exsolution techniques. Key areas requiring deeper investigation include the operational mechanisms of NPs compared to bulk grains, the development of methods for controlled NP fabrication, precise determination of the chemical composition of self-reconstructed NPs, and the long-term durability of NPs. A comprehensive understanding of NP growth and function within SOCs remains elusive and has yet to be fully validated experimentally. For example, infiltrated NPs may exhibit different operational mechanisms compared to those produced via exsolution, leading to distinct performance characteristics that require further scrutiny. In situ exsolved NPs, in particular, are the present subject of ongoing debates regarding ionic transport and electronic conduction between the anchored NPs and their parent materials. Although cell performance improvements have been observed with surface NPs, the underlying mechanisms driving these enhancements need to be experimentally elucidated, especially concerning accurate measurements of ionic diffusion and electronic conduction associated with the derived NPs. The controlled synthesis of NPs is crucial for advancing our understanding of intrinsic catalytic processes and improving overall cell performance. This involves synthesizing NPs with precise chemical compositions, creating NPs of specific sizes, and assembling NPs into unique morphologies. Current technologies primarily focus on successfully preparing NPs rather than fine-tuning their chemical makeup, dimensions, and shapes, which presents significant technical hurdles. Future developments should aim to create advanced methodologies for controlled NP fabrication. Theoretically, achieving the self-reconstruction of ultrasmall particles is feasible; however, in high-temperature SOCs, reducing exsolved NPs to cluster or even single-site metal active sites poses a practical challenge. Inaccuracies in determining the chemical composition of NPs hinder the evaluation of valuable information and complicate controlled and oriented NP synthesis. Although one-pot synthesis methods are widely used for in situ NP fabrication, accurately determining the chemical composition of NPs remains a formidable challenge. Developing NPs in SOCs necessitates multidimensional theoretical knowledge and experimental techniques. Post-exsolution, changes in the material matrix’s chemical composition can influence catalytic properties, an area that warrants reevaluation. Coherent effects arising from compositional changes in parent compounds and derived NPs should be explored, particularly the key mechanisms governing variations in catalytic properties, which require special attention. The long-term operational stability of NPs in SOCs is another unresolved issue. Under certain conditions, enhanced electrode catalytic activity comes at the cost of sustainability. Exposing more activated surfaces of NPs to harsh environments can compromise their long-term stability. Factors such as gas molecule adhesion and intermediate species dissociation can cause surficial lattice ions to deviate from their regular positions, disrupt surface symmetry, and accelerate secondary phase formation. As a result, catalysts containing active NPs face greater challenges in maintaining ultra-long-term stability. Reliable data from extended operation tests, such as thousands of hours of cell sustainability measurements, and long-term cycling tests are essential for assessing the true endurance capabilities of nano-based SOCs. Additionally, theoretically investigating the degeneration mechanisms of SOCs in the presence of NPs is an intriguing area of study.

In summary, while nanoparticle infiltration and metal exsolution have significantly improved SOC performance over the past decade, addressing these remaining challenges will be vital for future progress. This review aims to stimulate interest in common nanotechnologies within SOC research and provide valuable insights into cutting-edge fields. It also seeks to inspire new initiatives toward the commercialization of SOCs, paving the way for large-scale industrialization and economic feasibility in the near future.

## Data Availability

No primary research results, software or code have been included and no new data were generated or analysed as part of this review.
